# Extensive Cochleotopic Mapping of Human Auditory Cortical Fields Obtained with Phase-Encoding fMRI

**DOI:** 10.1371/journal.pone.0017832

**Published:** 2011-03-23

**Authors:** Ella Striem-Amit, Uri Hertz, Amir Amedi

**Affiliations:** 1 Department of Medical Neurobiology, The Institute for Medical Research Israel-Canada (IMRIC), Faculty of Medicine, The Hebrew University of Jerusalem, Jerusalem, Israel; 2 The Edmond and Lily Safra Center for Brain Sciences (ELSC), The Hebrew University of Jerusalem, Jerusalem, Israel; 3 The Cognitive Science Program, The Hebrew University of Jerusalem, Jerusalem, Israel; University of Leuven, Belgium

## Abstract

The primary sensory cortices are characterized by a topographical mapping of basic sensory features which is considered to deteriorate in higher-order areas in favor of complex sensory features. Recently, however, retinotopic maps were also discovered in the higher-order visual, parietal and prefrontal cortices. The discovery of these maps enabled the distinction between visual regions, clarified their function and hierarchical processing. Could such extension of topographical mapping to high-order processing regions apply to the auditory modality as well? This question has been studied previously in animal models but only sporadically in humans, whose anatomical and functional organization may differ from that of animals (e.g. unique verbal functions and Heschl's gyrus curvature). Here we applied fMRI spectral analysis to investigate the cochleotopic organization of the human cerebral cortex. We found multiple mirror-symmetric novel cochleotopic maps covering most of the core and high-order human auditory cortex, including regions considered non-cochleotopic, stretching all the way to the superior temporal sulcus. These maps suggest that topographical mapping persists well beyond the auditory core and belt, and that the mirror-symmetry of topographical preferences may be a fundamental principle across sensory modalities.

## Introduction

Vision, audition and touch are characterized by a topographical mapping of the sensory world onto the peripheral sensory epithelia (retinotopic, cochleotopic and somatotopic mapping), which is maintained along the pathway (e.g. thalamus and other brainstem nuclei) all the way into the primary sensory cortices. The prevalent view is that in higher-order sensory areas, such maps are gradually lost in favor of more complex or abstract representations. This view has recently been refined in the visual system [1], [2], [3], in which higher-order processing regions were shown to have clear retinotopic preferences in addition to their selectivity to complex visual features. For example, the parahippocampal place area (PPA) shows selectivity for place stimuli (such as pictures of houses) and a peripheral retinotopic eccentricity preference, and the fusiform face area (FFA) has a combined preference for faces stimuli and a foveal retinotopic eccentricity preference [2]. Recent studies by several groups showed that new spatial fields can be found not only in areas in the visual (occipital) cortex previously considered non-retinotopic [2] but even in the parietal and prefrontal cortices [3], [4]. Both in early and higher-order areas, spatial-retinotopic mirror-symmetry reversal maps have proved to be extremely useful in defining the borders between visual areas (from V1 and V2 and up to V7/V8 and the new fields in the parietal and prefrontal cortex) and the hierarchy of the visual system in general [5], [6].

In contrast to the visual domain, relatively little is known about the cochleotopic (i.e. tonotopic) organization in the *human* auditory cortex in general and beyond the primary core areas in particular. The structural anatomical division of the auditory cortex had been very thoroughly studied [7], [8], [9] in human and non-human primates (and well as in non-primates). These studies have divided the temporal auditory cortex to multiple fields based on cytoarchitectonic and chemoarchitectonic markers, and showed that the auditory cortex may be divided [10], [11], [12], [13], [14], [15], [16] to a koniocortical core area and which was further extended and divided functionally and anatomically [10], [13], [17], [18], [19] to three core areas, A1, R, and RT (and which may be further anatomically divided to multiple fields [20]). Surrounding it is a belt of smaller areas in the medial (also referred to as root [20]) and lateral aspects of the core (divided in primates and humans to at least 7 or 8 fields [15], [21]), an additional area of lateral parabelt regions (anatomically divided to a caudal and a rostral field [15]), and other high-order auditory fields extending to the caudal temporal plane and parietal operculum [10], [22], [23], [24]. These structures have been identified in non-human and human primates, and while they somewhat vary in position, size and architectonic appearance across taxonomic groups (such as relatively larger volume of the core relative to the belt in humans, but not in monkeys, and in enlargement of area Tpt in the human [20], [23]), they can nonetheless be identified as homologous structures [16], [20], [25]. Furthermore, auditory processing continues to the frontal and parietal cortices, in a highly specified connectivity pattern [26], [27], [28], in which, for example, the dorsolateral prefrontal cortex is accessed by the caudal aspect of the auditory belt and parabelt (also through connectivity to posterior parietal cortex) and the rostral and ventral frontal lobe are connected to the anterior belt and parabelt region. However, despite extensive years of research, the functional division of this vast auditory network has been lacking (especially in humans), in part due to incomplete mapping of functional markers such as cochleotopic borders between these areas [29]. Studies in primates [7], [16], [17], [18], [19], [21], [30] and other mammals [31], [32], [33] have investigated areas somewhat beyond the auditory core, and defined multiple cochleotopic maps in the auditory belt. Specifically, it was demonstrated that the core areas A1, R and RT contain cochleotopic mapping, with a low-frequency border dividing areas A1 and R, and a high-frequency border dividing R and RT. The belt fields seem to show cochleotopic gradients continuous with those of the core, apart from area CL, which shows a distinct cochleotopic gradient, generating an additional mediolateral high-to-low frequency gradient posterior to A1 [34]. In humans only several cochleotopic mapping works have been conducted, and these suggest that topographic mirror symmetry organization is present in the core auditory areas around Heschl's gyrus (thus referring to human homologues of areas A1 and R [8], [35], [36], [37], [38], [39]), and several studies [37], [40], [41] also looked beyond the auditory core to larger parts of the superior temporal plane, and reported frequency-dependent response regions, or cochleotopic gradients which may correspond to some of the auditory belt areas. However, these studies (both in humans and non-humans) only examined a limited part of the auditory cortex which did not cover the entire higher-order auditory areas in the temporal lobe (for example the parabelt areas) or beyond it.

Outside the auditory core, and even more so outside the auditory belt, in the parabelt regions and in auditory regions outside the temporal lobe, fidelity to cochleotopic organization is thought to deteriorate greatly [29], [42]. This makes it much more difficult to define the borders and number of these auditory regions in humans, and thus also to distinguish them functionally, and to compare findings across groups (especially when compared to the very well defined human visual retinotopic areas). These regions are, in general, considered higher-order auditory areas, and are thought to be driven mostly by more complex auditory features and stimuli (both in humans and in other mammals) such as spatial location, source identity, pitch and melody and different types of object sounds, species-specific vocalizations, or speech rather than by pure tones [43], [44], [45]. Recent studies have indicated that even A1 does not only show frequency sensitivity but also partakes in relatively complex analyses such as selective responses to combinations of auditory attributes or auditory objects [46], and that belt areas may show evidence of multisensory integration [47]. However, it is important to note that these options (cochleotopic or more complex), preferences or receptive fields characteristics are not mutually exclusive, just as visual object related areas can show both object category and retinotopic preferences (e.g. face and foveal in FFA and places and peripheral in PPA). Taken together, these pieces of information paint a somewhat limited picture of human auditory cortical processing in relation to its cochleotopic or tonotopic organization (as noted in several recent reviews, e.g. [29], [42]). Better understanding of cochleotopic organization of human auditory cortex (especially if organized in mirror symmetry organization) can greatly help in parceling of the high-order auditory cortex into functional units, which can then be integrated in a more general model of the auditory system within the framework of current developing putative models (e.g. the two processing streams model for different auditory functions [7], [26], [48], [49], [50], [51]).

Here, we set out to study the cochleotopic preferences of the entire human cerebral cortex, in order to answer the following questions: 1. how many cochleotopic maps are there in the human cerebral cortex? 2. Is cochleotopic preference indeed limited to the auditory core and belt areas or does it extend to the higher-order parabelt regions around the superior temporal sulcus, and even beyond them to higher order auditory areas? 3. Are these areas arranged in a mirror-symmetry organization, enabling their putative parceling to auditory fields, similarly to the visual cortex? 4. If so, can we generalize the large-scale governing principles of organization regarding multiple topographical representations which are sensory modality invariant? Are the entire visual **and** auditory cortices, in addition to other functional sensory specificities, fundamentally topographical in nature?

**Figure 1:**
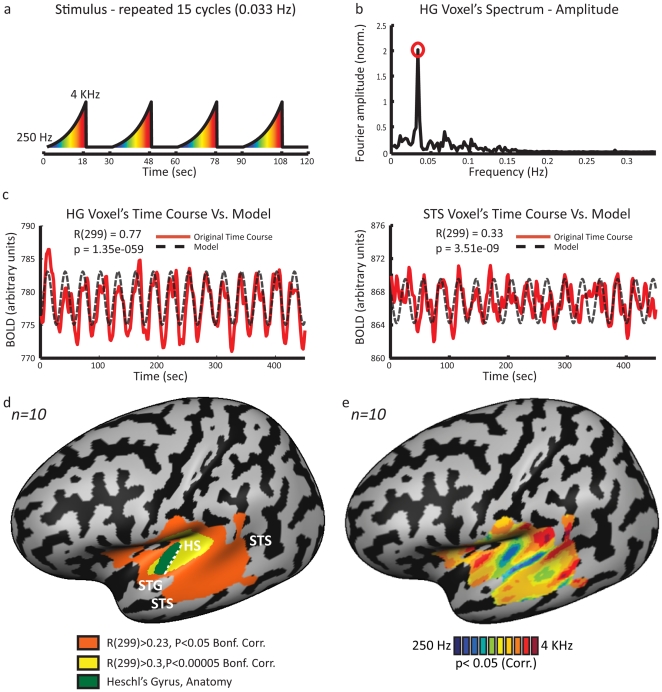
Experimental design and spectral analysis. **A.** Stimulus – the subjects heard a dynamic ascending pure tone chirp, which repeated 15 times (stimulus repetition frequency 0.033 Hz). **B.** Each voxel's time-course was Fourier transformed. Presented here is the normalized amplitude of the spectrum of a voxel sampled from Heschl's gyrus (HG) of a representative subject. Amplitude at stimulus repetition frequency is marked with a red circle. The voxel's phase at that frequency corresponds to the preferred tone (auditory frequency) of the voxel. **C.** Amplitude and phase parameters were used to construct a pure cosine used as a model of the activation. The original raw time-course of two voxels, one from HG and one from the superior temporal sulcus (STS) are drawn in red; the dashed black line shows the model for each voxel. Pearson correlation coefficients were calculated to estimate the significance of the response of each voxel, and phase maps were inspected only in regions showing high and significant correlations. **D.** Mean correlation coefficient (Pearson's R) map of 10 subjects, presented on a partly inflated left cortical hemisphere of the standard MNI brain. Most of the auditory cortex is marked with high R values (marked red, R(299)>0.23, p<0.05 Bonf. Corr.). Within this region R values are the highest in the core area (marked in yellow, R(299)>0.3, p<0.00005 Bonf. Corr.), including HG (marked in green) and its surroundings. For a presentation of Pearson's R values in a horizontal slice view see **Fig. S2C**. HS – Heschl's sulcus, STG – Superior temporal gyrus, STS – Superior temporal sulcus. **E.** Group (Session 1, *n = 10*) relative frequency preference map is presented in a lateral view of the partly inflated left cortical hemispheres of the standard MNI brain. The map within the auditory-responsive region shows multiple iso-frequency bands, in addition to the mirror- symmetric cochleotopic maps in the auditory core area on the superior temporal plane. These iso-frequency bands extend in a superior-to-inferior axis along the temporal cortex to the superior temporal sulcus.

Using functional magnetic resonance imaging (fMRI) we studied ten subjects while they listened to a logarithmically rising tone chirp spanning the range of 250–4,000 Hz in 18 seconds (**Fig. 1A**). We then applied an in-house modified version of spectral analysis techniques [32], [52], [53] to study the frequency sensitivity of the human cerebral cortex.

## Materials and Methods

### Subjects

Ten healthy subjects (4 females) aged 24–35 participated in the experiment. The Tel–Aviv Sourasky Medical Center Ethics Committee approved the experimental procedure and written informed consent was obtained from each subject.

### Stimuli and experimental protocol

For the main experiment (Exp. 1) subjects were presented with a rising logarithmic tone chirp spanning the range of 250–4,000 Hz in 18 seconds, followed by a 12 second baseline period with no auditory stimulation. Tones in higher and lower frequencies (though perceivable by humans) were not used in the current setting to avoid distortion of high frequency sounds inside the scanner and due to other limitations of our system. The chirp onset was ramped using a 20 ms logarithmically rising envelope to avoid an attention bias to the loud sound onset and widespread and non-specific auditory activation. This was another advantage (in addition to greater sensitivity for continuous relative mapping, see below) in using continuous stimulation rather than short chirps (separated by silent periods) each in a different frequency band. This 30 second cycle was repeated 15 times, resulting in a presentation frequency of 0.033 Hz. In addition there was a 30 second period of silence before and after the 15 cycles of auditory stimuli (**Fig. 1A**), used as baseline measurements.

Half the subjects were also scanned again in an additional control experiment (Exp. 2), in which the frequency modulation was in the opposite direction (i.e. beginning in 4 KHz and ending in 250 Hz, falling chirp), to preclude apparent tonotopic gradients resulting from the direction of the frequency modulation. In order to inspect test-retest reliability of our results, a subgroup of four subjects was scanned again on the main experiment in a different day (Exp. 3).

Subjects wore blindfolds and had their eyes shut for the duration of the scan, in order to focus on the auditory stimulus, and were instructed to listen carefully to the sounds. The stimulus was presented to both ears using fMRI-compatible electrodynamic headphones (MR-Confon, Germany), specifically designed to reduce scanner noise, which were needed as the scanner noise is not equal across auditory frequencies [54] and may add biases in cochleotopic mapping. However, measures were taken to prevent the stimulus being masked by the scanner noise. Each subject heard the basic chirp inside the scanner prior to the experiment, with all insulations in place and while the scanner was working. This was done in order to make sure subjects could hear the entire chirp clearly on top of the scanner noise. Stimulus intensity was set individually at levels between 86–89 dB SPL in order to optimize hearing on top of scanner noise. The intensity was kept constant across frequencies, so overall RMS level was equal to the individual dB SPL. The continuous nature of the auditory stimulus and our data analysis techniques are not optimal for sparse sampling approaches to data acquisition, so the stimulus had to exceed scanner noise (see also above, due to non-specific auditory activation in sparse presentation). The limitations of the auditory devices in the noisy scanner environment constituted a restriction on the cochleotopic mapping. Auditory neurons tend to show frequency selectivity only near the perceptual threshold, while at relatively high sound intensities auditory filters are much broader [55], and show more moderate frequency sensitivity. However, due to the advantages of using a continuous stimulus (which, at least in retinotopy, greatly increases the sensitivity of retinotopic mapping), and since higher order auditory areas are more sensitive to chirps (see below), we chose to present the auditory chirp well above the individual hearing threshold. Additionally, presenting the stimuli at high intensities had the advantage of maximally activating the entire auditory cortex, including non-core regions which are not strongly driven by tones [34], [45], [56], but may still show widely-tuned frequency selectivity [30], [57], and areas responsive to threshold best frequencies higher than 4 KHz. This is an additional advantage to spectral analysis, which compares the auditory response to a wide cosine wave, supporting the inclusion and inspection of widely-tuned neuronal populations, as opposed to previously used GLM approaches. However, it is possible that in future studies using lower-intensity stimuli further mapping might be more crisp and accurate. Thus, the several novel mirror symmetry cochleotopic maps reported here (see results) might even be an underestimate of the total number of topographical cochleotopic areas in the cerebral cortex.

### Functional MRI acquisition

The BOLD fMRI measurements were obtained in a whole-body, 3–T Magnetom Trio scanner (Siemens, Germany). The fMRI protocols were based on multi-slice gradient echoplanar imaging (EPI) and a standard head coil. The functional data were collected under the following timing parameters: TR = 1.5 s (relatively short TR to better fit the temporal resolution needed for the phase-locking Fourier approach used here), TE = 30 ms, FA = 70°, imaging matrix = 80
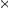
80, FOV = 24
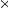
24 cm (i.e. in-plane resolution of 3 mm). 22 slices with slice thickness = 4.5 mm and 0.5 mm gap were oriented in the axial position, for complete coverage of the whole cortex. We chose to scan the entire brain despite the tradeoff with scan resolution so as to map cochleotopic fields beyond the well-known areas in superior temporal plane, both in higher-order regions of the temporal auditory cortex, and outside the temporal lobe, similar to the visually-topographic maps found in the parietal and frontal lobes [3], [4].

### 3D recording and cortex reconstruction

Separate 3D recordings were used for coregistration and surface reconstruction. High resolution 3D anatomical volumes were collected using T1-weighted images using a 3D-turbo field echo (TFE) T1-weighted sequence (equivalent to MP-RAGE). Typical parameters were: Field of View (FOV) 23 cm (RL)×23 cm (VD)×17 cm (AP); Foldover- axis: RL, data matrix: 160×160×144 zero-filled to 256 in all directions (approx 1 mm isovoxel native data), TR/TE = 9 ms/6 ms, flip angle = 8°.

Group results were superimposed on a cortical reconstruction of the standard MNI (Montreal Neurological Institute) brain, which was transformed to Talairach coordinates [58]. Cortical reconstruction included the segmentation of the white matter using a grow-region function embedded in the Brain Voyager QX 1.9.10 software package (Brain Innovation, Maastricht, Netherlands). The cortical surface was then inflated to expose the hidden sulci.

### Data Analysis

We analyzed the data in our experiments using several complementary methods of analysis. These included individual subject analyses to verify the between-subject between-scans repeatability of the results, and group analyses to extend the findings to the population level. In individual subjects we examined the individual spectral maps (see the details of this analysis below in section *I. Spectral and linear correlation analysis*), as well as individual cross-correlation (section *III. Cross correlation analysis*) and raw time-course event-related averaging analysis (section *V. General Linear Model analysis*). For the group-level analyses, we looked at the group results in the spectral maps (section *II. Group analysis*), in a cross-correlation analysis (section *III. Cross correlation analysis*) in GLM random effect maps and in raw time-course event-related averaging analyses (section ***V.*** *General Linear Model analysis*). Additionally, in order to further assess the test-retest reliability of our results and avoid stimulus order confounds, we compared the results obtained in the main experiment (Exp. 1) with those of the two control experiments (Exp. 2–3) via spectral maps, cross-correlation, GLM and time-course analysis, and additionally applied an objective quantitative similarity analysis (section *IV. Map alignment measure*).

Prior to these extensive analyses, preprocessing data analysis was performed using the Brain Voyager QX 1.9.10 software package. This involved removal of the first eight images (during the first baseline rest condition) because of non-steady state magnetization. Functional MRI data preprocessing also included head motion correction, slice scan time correction and high-pass filtering (cutoff frequency: 3 cycles/scan) using temporal smoothing in the frequency domain to remove drifts and to improve the signal to noise ratio. All data included in the study did not exceed motion of 2 mm in any given axis, nor did it have spike-like motion of more than 1 mm in any direction. Functional and anatomical data sets for each subject were aligned and fit to standardized Talairach space [58]. General Linear Model Analysis and cross-correlation analysis were also conducted using the Brain Voyager QX 1.9.10 software package (for details see below). All additional analyses were conducted using analysis code developed in the lab on MATLAB (MathWorks, Natick, MA) and then imported back onto Brain Voyager to display on the Talairach normalized volume anatomical recording of the MNI brain or individual brain, or on the inflated MNI cortical surfaces.

### Data Analysis I. Spectral and linear correlation analysis

Following standard retinotopy procedures [52], [53], [59], [60] and auditory mapping in mammals [32], [61], we applied Fourier analysis to the auditory responses locked to the stimulus repetition frequency (with some modifications, see details below and in **Fig. 1B**). Prior to frequency analysis, time-courses were de-trended to remove mean and linear drifts. The complex Fourier at the repetition frequency 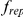 is denoted by:
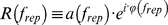
(1)where 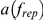 represents the amplitude and 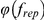 the phase, and calculated by:
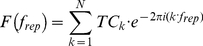
(2)where *TC* represents the sample time-course, and *N* is the number of sampled time points (300).

Following Engel and colleagues [52], both amplitude and phase parameters were used to construct a pure cosine serving as a model of the activation (**Fig. 1C,D** **,** ***Eq. 3***). A Pearson correlation coefficient was then calculated between the model and the original time-course. This procedure yielded a correlation coefficient for each voxel. This correlation coefficient can also be written as a normalized Fourier coefficient:
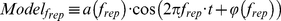
(3)

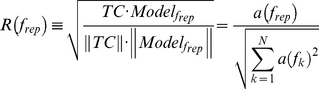
(4)The correlation coefficient was used as a direct measure of the voxel's response to the auditory stimulus. The correlation coefficient (R) was transformed (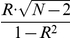) and used as t statistic with N−2 degrees of freedom (in our case N = 300), to calculate, independently for each voxel, the significance of the cortical response to the auditory stimulus.

In regions showing high correlation to the stimulus repetition frequency, the phase value was extracted from the complex coefficient (**Eq. 1**) according to:
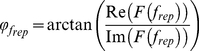
(5)The phase corresponded to the latency of the voxel's response to the chirp, which, if each voxel has tonal selectivity (resulting from the tuning curves of the neurons in that voxel), should correspond to the preferred tone (auditory frequency) of that voxel. Phase values were distributed between −π and π, and were linearly transformed to range between 0 and 30, representing time points in each stimulus cycle. Due to the time delay of the hemodynamic response, the phase code does not temporally overlap with the stimulus presentation time. The phase onset of the response detected in the anatomically defined Heschl's gyrus (as well as its bordering sulci; at an average of 6.3±1.6 seconds after stimulus onset, in accordance with standard hemodynamic delay [62]) was considered to represent the response to a tone frequency of 250 Hz, which was the first frequency in the chirp. Similarly, the latest response (last preferred phase) observed in Heschl's gyrus surroundings (an average of 23.76±2.5 seconds after stimulus onset) was assumed to correspond to a tone frequency of approximately 4 KHz, the last tone frequency presented. Latencies between the first and last responses were interpreted as deriving from intermediate tone frequencies progressing from lower to higher frequencies (note that we do not intend to define the exact best frequency of each voxel, but rather the relative preference to a tone range, be it high, medium or low frequency range). This resulted in an individual response range, cropped according to the individual onset and offset of hemodynamic delay in responses of the auditory cortex. The average hemodynamic response duration for the group was 17.46 seconds, matching the stimulus duration (18 second), thus verifying the validity of the response ranges. These values constructed the phase code corresponding to the relative preferred tone frequency of each voxel, and resulted in individual phase code maps which correspond to individual relative frequency preference maps. While the latency could potentially also signify a lagged response due to intracortical processing in higher-order auditory cortex regions, the entire analysis of an auditory stimulus in the cerebral cortex would not typically require more than a second (for example see [48]); thus it is unlikely to manifest in belated responses in the time scale of many seconds, such as the length of the auditory stimulus. Moreover, should an entire region receive the information at a later time without having an inner cochleotopic mapping, no difference in tone sensitivity should be seen within this region.

### Data Analysis II. Group analysis

Single subject correlation coefficient maps were spatially smoothed with a three dimensional 6 mm half width Gaussian in order to reduce inter-subject anatomical variability, and then averaged to create a group averaged correlation coefficient map. The average correlation coefficient map was statistically assessed in fixed effect model analysis. Specifically, in the group results, voxels that were characterized with high correlation coefficients across subjects also demonstrated high between- subject variability, compared with voxels with low correlation coefficient values. This was due to an uneven distribution between zero and one of correlation coefficients, making between-subject analyses (similar to random effect analysis in GLM, which was applied as a supplementary analysis, see below) not appropriate in our case. Voxels whose correlation coefficient satisfied a predetermined statistical threshold were chosen as a mask, and the group average phase values were computed within that mask. Due to differences in hemodynamic delay between subjects, the response range was different for different subjects. In order to normalize the response range across subjects, average initial response and final response points were calculated (as detailed above) and each subject's phase code was linearly transformed to range between them. Individual subject spectral analysis is presented at a significance threshold of *p*<0.05. Significance levels were calculated taking into account the probability of a false detection for any given cluster [63], thereby correcting for multiple comparisons across all voxels. For group analysis, the native resolution transformed phase maps were spatially smoothed with a three dimensional, 4 mm half width Gaussian, and averaged to create a mean phase map. These maps' response range was narrowed because of the averaging procedure, and was rescaled in the same manner as the single subject maps, according to primary auditory cortex initial and final phase. This procedure yielded group phase maps that only display voxels with a phase within the group response range (phase code), masked to be displayed only in voxels whose average correlation coefficient (R) values exceeded a predetermined statistical threshold of p<0.05 strictly corrected for multiple comparisons using the Bonferroni correction according to the number of voxels in the cortex.

In order to account for the hemodynamic response delay directly, without the possible confound of the interpolation in the creation of response ranges, we also directly averaged the preliminary phase maps derived from chirps moving in opposite directions similarly to [52], [53] who used the same approach in retinotopic studies (n = 5: rising chirp in Exp. 1 and falling chirp in Exp. 2). This averaging cancels the phase delays resulting from the hemodynamic response function (HRF), as the HRF is expected to delay the response in opposite directions in the two scans.

To further supplement phase analysis and to address possible general confounding factors, such as compensating for comparing the hemodynamic signal to cosine function (Fourier analysis), which differs from the delayed typical hemodynamic response function [64], we also conducted supplemental cross-correlation and General Linear Model analyses in both group level and single subject level (see below), as well as applied a statistical map alignment measure to quantitatively compare the results of the different analyses and experiments.

### Data Analysis III. Cross correlation analysis

As a complementary analysis, we applied a standard cross-correlation analysis using the Brain Voyager QX 1.9.10 software package to the individual time-courses following preprocessing steps only. We used the predicted standard hemodynamic signal time-course for the first 1/12 of a stimulation cycle (1 TR, 1.5 seconds) and shifted this reference function successively in time (time steps corresponded to the recording time for one volume, TR). Sites activated at particular ranges of tones were identified through selection of the lag value that resulted in the highest cross-correlation value for a particular voxel. Individual subject cross-correlation analysis is presented at a significance threshold of *p*<0.05. Significance levels were calculated taking into account the probability of a false detection for any given cluster [63]. Group analysis was conducted on the averaged data of the individual subjects for each experiment.

### Data Analysis IV. Map alignment measure

In order to quantify the compatibility between the different cochleotopic maps we used an alignment index introduced by Sereno and Huang [3]. This measure was used to compare the group phase maps, i.e. rising chirp, falling chirp and returning rising chirp group. We also used it to compare the replicability of tonotopic pattern across subjects. Alignment index was calculated voxel-wise, defined as
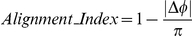
Where 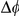 is the difference between the phases of two voxels (in radians). This index is 1 when the phases are identical across the maps, and reaches 0 when the phases are opposite one another. The similarity of two maps can be therefore evaluated by comparing the distribution of its alignment indexes to that of random maps. If the maps are similar, alignment indexes will distributed with a sharp peak towards 1. Random maps indexes are distributed with linear increase towards 1 (see [3] for further details). Random distribution was marked on the histograms of the group maps alignment indexes with red line for comparison. We tested the diversion from random distribution statistically by using t-test between two groups to get a p value. When testing single subjects' maps replicability, a pair-wise comparison between each map pairs was conducted, resulting with a matrix of alignment indexes. Each was compared with random distribution, as well as the average of all indexes.

### Data Analysis V. General Linear Model analysis

In order to assess the tone selectivity using an independent supplementary analysis, the continuous auditory stimulus was divided into low, medium and high frequency tones (250–1000 Hz, 1–2.25 KHz, 2.25–4 KHz; lasting 4 TRs or 6 seconds each) periods, which were used as conditions in a block design protocol. Predictors for a general linear model (GLM) were built by convolving the auditory conditions with a typical hemodynamic response function [64]. GLM maps present the contrast of each of these tone predictors compared to the other predictors (e.g. high frequency vs. low and medium frequency tones), at a *p*<0.05 threshold (corrected for multiple comparisons [63]). The average time-course of activation for individual subjects was sampled from peaks of iso-frequency bands, and averaged at the time of peak hemodynamic response (4.5–7.5 seconds after the frequency bin stimulus onset, TRs 3–5) to extract the average percent signal change. In the group analysis, across-subject statistics were calculated using a hierarchical random effects model [65] allowing for generalization of the results to the population level. The average time-course of activation was sampled and averaged to extract the average percent signal change. The standard error was also calculated across subjects and is displayed in the error bars. This analysis, though less optimal for continuous stimuli, serves as a way to acquire the **raw** averaged percent signal change and to illustrate the frequency preferences of iso-frequency bands, as determined by the phase code maps.

## Results

In order to examine our results in different ways and validate them, we applied several complementary methods of analysis. These included individual subject analyses to verify the between-subject between-scans repeatability of the results, and group analyses to extend the findings to the population level. In individual subjects, we examined the individual phase maps, displayed both on the anatomical recording of each subject's auditory cortex and on the inflated cortical sheet, as well as individual cross-correlation and raw time-course analysis. For group-level analyses, we looked at the group results in the phase maps on the 3D brain recording, on the inflated cortical sheet, in a cross-correlation analysis, in GLM random effect map and averaged raw time-course analyses of the entire cortex. This helped us test how many cochleotopic maps there are in the human cerebral cortex within and outside the auditory core and belt areas and to examine the putative mirror-symmetry organization of these cochleotopic maps. Additionally, in order to further assess the test-retest reliability of our results and avoid stimulus order confounds, we also scanned a subgroup of the original subjects in a falling chirp control experiment (Exp. 2), and a subgroup of subjects in a second repetition of the main experiment (Exp. 3), and compared the results obtained via phase maps, cross-correlation, GLM and time-course analysis to that of the main experiment (Exp. 1).

For our main analysis method, we adapted spectral analysis ([52], [53], see full description in methods section) to extract the correlation coefficient of each voxel's response to a model of the auditory stimulus repetition frequency and its phase (see **Fig. 1**). The correlation coefficient (*R*) was used as a statistic to calculate the significance of the cortical response to the auditory stimulus. The minimum significance level was set to *P*<0.05, strictly corrected for multiple comparisons using the Bonferroni correction. An area covering vast parts of the temporal lobe showed a highly significant correlation to the auditory stimulus repetition frequency, and the most strongly correlated area was located in the auditory core areas (Heschl's gyrus and its surroundings; **Fig. 1D** **, Figs. S1, S2C**). Each voxel within the responsive area was assigned a color representing the phase of the response, which, as sound frequency varied systematically with time during the auditory stimulus, was indicative of the preferred tone (see **Fig. 1**) of that voxel.

As our aim was to look for broad cochleotopic mapping in the entire human cortex, even outside the “traditional” auditory cochleotopic cortex within the temporal lobe, we initially inspected several regions which showed significant responses to our stimulation protocol; i.e. a highly significant correlation coefficient in all three experiments (**Fig. S1**). These regions included bilateral activation in the posterior-inferior frontal lobe, medial superior frontal gyrus\premotor cortex, precuneus, and a left inferior parietal cluster, regions sporadically reported previously to be involved in various auditory localization and recognition tasks [48], [50], [77], [78]. However none of these regions showed a clear and consistent full cochleotopic arrangement across the experiments and between the subjects. Hence, we focused our attention on the temporal lobe, which showed robust, extensive and reliable responses, stretching all the way from Heschl's gyrus to the superior temporal sulcus.

**Figure 2:**
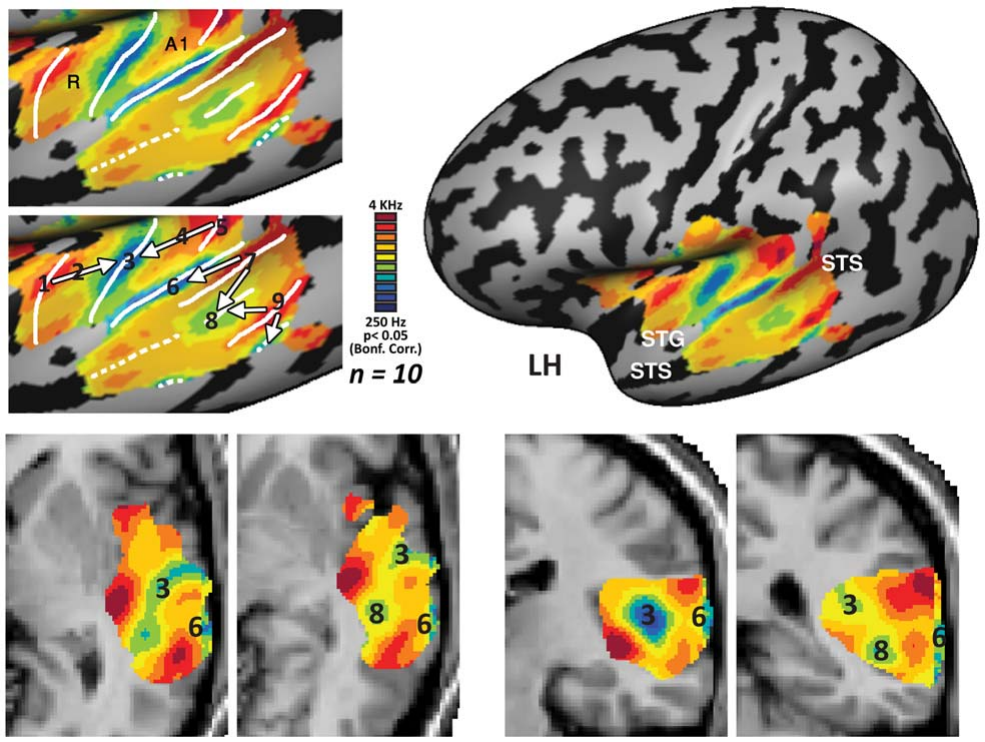
Multiple mirror-symmetric cochleotopic maps in the left hemisphere of the human auditory cortex. Group (Exp. 1, *n = 10*) relative frequency preference map is presented in a lateral view of the inflated left cortical hemisphere of the standard MNI brain, exposing the entire cochleotopic organization of the multiple iso-frequency bands (STG – Superior temporal gyrus, STS – Superior temporal sulcus). On the left panels, the auditory cortex region is magnified, showing Exp. 1 relative frequency preference map on the cortical surface. The estimated border between the putative mirror- symmetric cochleotopic maps is indicated (white line) in the lowest and highest frequency tones which represent the mirror-symmetry flipping lines between the homologues of A1 and R in the core auditory cortex, and between multiple additional cochleotopic fields. Numbers indicate points along the cochleotopic gradients (similar to those depicted in **Fig. 6**, from which raw time-courses of activation were sampled), with white arrows demonstrating the gradient direction in each filed. On the lower panels, the same gradients are depicted in volume views in horizontal (z = 2, −1), and coronal (y = −21, −32) slices, numbered similarly to the surface view (for demonstrative and orientation purposes only), to enable the identification of the same gradients in the three-dimensional based views.

**Figure 3 pone-0017832-g003:**
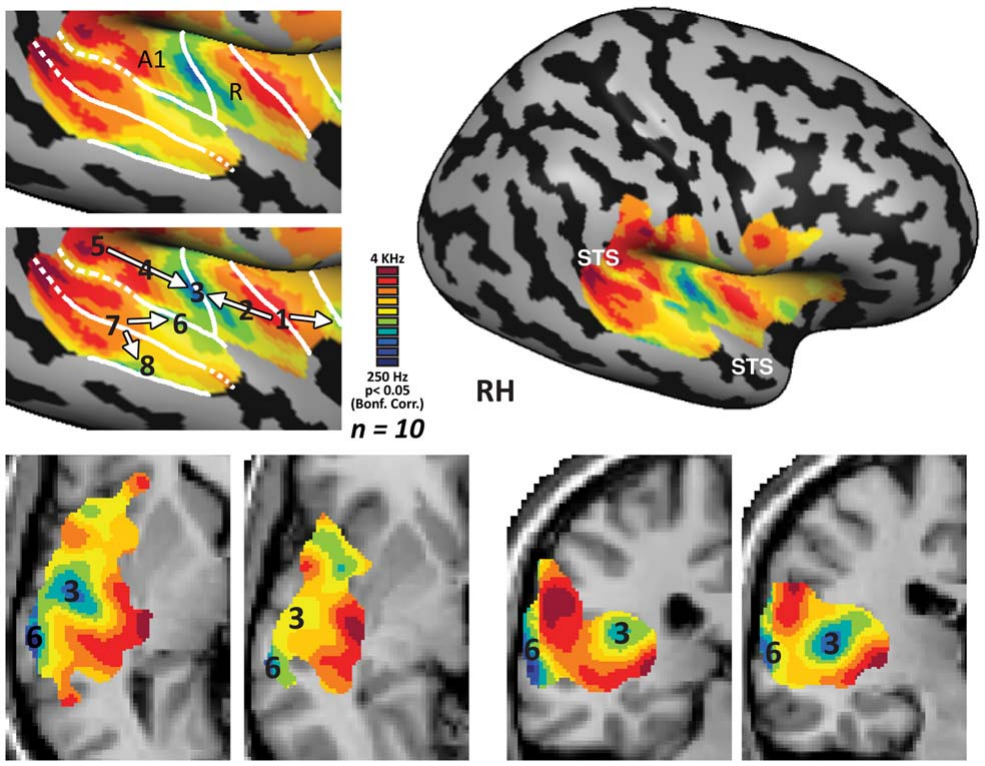
Multiple mirror-symmetric cochleotopic maps in the right hemisphere of the human auditory cortex. Group (Exp. 1, *n = 10*) relative frequency preference map is presented in a lateral view of the inflated right cortical hemisphere of the standard MNI brain, exposing the entire cochleotopic organization of the multiple iso-frequency bands (STG – Superior temporal gyrus, STS – Superior temporal sulcus). On the left panels, the auditory cortex region is magnified, showing Exp. 1 relative frequency preference map on the cortical surface. The estimated border between the putative mirror- symmetric cochleotopic maps is indicated (white line) in the lowest and highest frequency tones which represent the mirror-symmetry flipping lines between the homologues of A1 and R (and possibly, anterior to it, RT) in the core auditory cortex, and between multiple additional cochleotopic fields. Numbers indicate points along the cochleotopic gradients (similar to those depicted in **Fig. 6**, from which raw time-courses of activation were sampled), with white arrows demonstrating the gradient direction in each filed. On the lower panels, the same gradients are depicted in volume views in horizontal (z = 4, 0), and coronal (y = −28, −22) slices, numbered similarly to the surface view (for demonstrative and orientation purposes only), to enable the identification of the same gradients in the three-dimensional based views.

The cochleotopic organization of the core auditory areas was highly replicable across individual subjects (see **Fig. S2A** for **unsmoothed** tone-preference maps of all 10 subjects) and highly consistent with previous studies. Most (9/10) single subjects clearly displayed a topographical mapping pattern of tone-preference shift from high-frequency tones to low-frequency tones and back along the superior temporal plane with Heschl's gyrus (HG) located within this mirror-symmetric large scale organization. This is highly consistent with the general pattern found in primates [30] and in recent neuroimaging studies in humans [35], [38], [40], [41]. Thus our results confirm the suggestion of Formisano and colleagues (2003) that this mirror-symmetric mapping corresponds to the human analogues of the core auditory areas A1 and R. This large scale cochleotopic organization pattern was present in both hemispheres (**Figs. 2** **,** **3**). However, in the right hemisphere we found another putative anterior mirror-symmetry map resulting in a possible large scale organization of high-low-high-low (see **Fig. 3** for inflated and horizontal views, and the sagittal view in **Fig. S2B**). This additional map is also in general agreement with the organization of primate core areas [30], and may correspond to a human analogue of area RT. Some of our single subjects (6/10) also showed evidence of a medial-lateral cochleotopic gradient on medial HG (see **Fig. S2A**) [39], [66]. However, this gradient was not as consistent as the large scale high-low-high frequency mirror- symmetric pattern (again consistent with human imaging findings [35], [38], [41]). This could be ascribed to a different of orientation of the gradients in humans (see discussion for details of the contemporary controversy in the matter) or to the high variability of the position and extent of the primary auditory cortex in relation to the location of HG [8], [67]. Alternatively, this could be due to our limited resolution, a problem which could be resolved in future studies focusing on HG with higher spatial resolution and also possibly using higher-field scanners (for example, see [30], which used a 7-Tesla MRI scanner).

As observed in most individual subjects, group analysis of cochleotopic selectivity indeed showed large scale high-low-high frequency mirror- symmetric cochleotopic maps in the core auditory cortex of humans (**Figs. 1E** **,** **2** **,** **3** **, Fig. S2B**; see also **Movie S1** depicting the propagation of frequency sensitivity).

While the peak of the correlation to the auditory stimulation was located in the primary auditory cortex region, the significantly responsive area (**Fig. 1D**, **Fig. S2C**, p<0.05, Bonf. corrected) extended well beyond core areas all the way to parts of the superior temporal sulcus, and parts of the middle temporal gyrus, regions considered to be higher-order auditory and multisensory cortices responsive to complex sounds or even multisensory integration areas. A phase analysis of these areas revealed gradients between multiple bands of tone frequency selectivity (see **Figs. 1E** **,** **2** **,** **3** – on both cortical and volume views). These large-scale mirror-symmetric tone-selective bands extended along the superior-to-inferior axis to the superior temporal sulcus. Although both hemispheres exhibited at least two new mirror symmetry maps with a superior-inferior axis, there are indications that there are additional maps extending as far as the middle temporal sulcus in the left hemisphere (see relative frequency preference map in **Figs. 2** **,** **3** and in further analyses below; see also **Movie S1**). In order to assess the number and location of the possible auditory fields, we delineated (**Figs. 2** **,** **3**, left upper panel) putative cochleotopic map borders according to preferences for the lowest and highest frequency tones which represent the mirror-symmetry flipping lines, and marked the approximate gradient seen between such flipping lines (marked in arrows and numbers in **Figs. 2** **,** **3**, left middle panel). Despite the reliance on the tone extremities for the auditory field parceling, it is important to note that the entire stimulus tone range, with a gradual shift in the cortical preference, is represented in the mapping (e.g. **Movie S1** and note the middle tone frequencies in the GLM analysis below). These novel cochleotopic maps were also consistent across subjects with certain expected variability (**Figs. S3, S4**).

**Figure 4 pone-0017832-g004:**
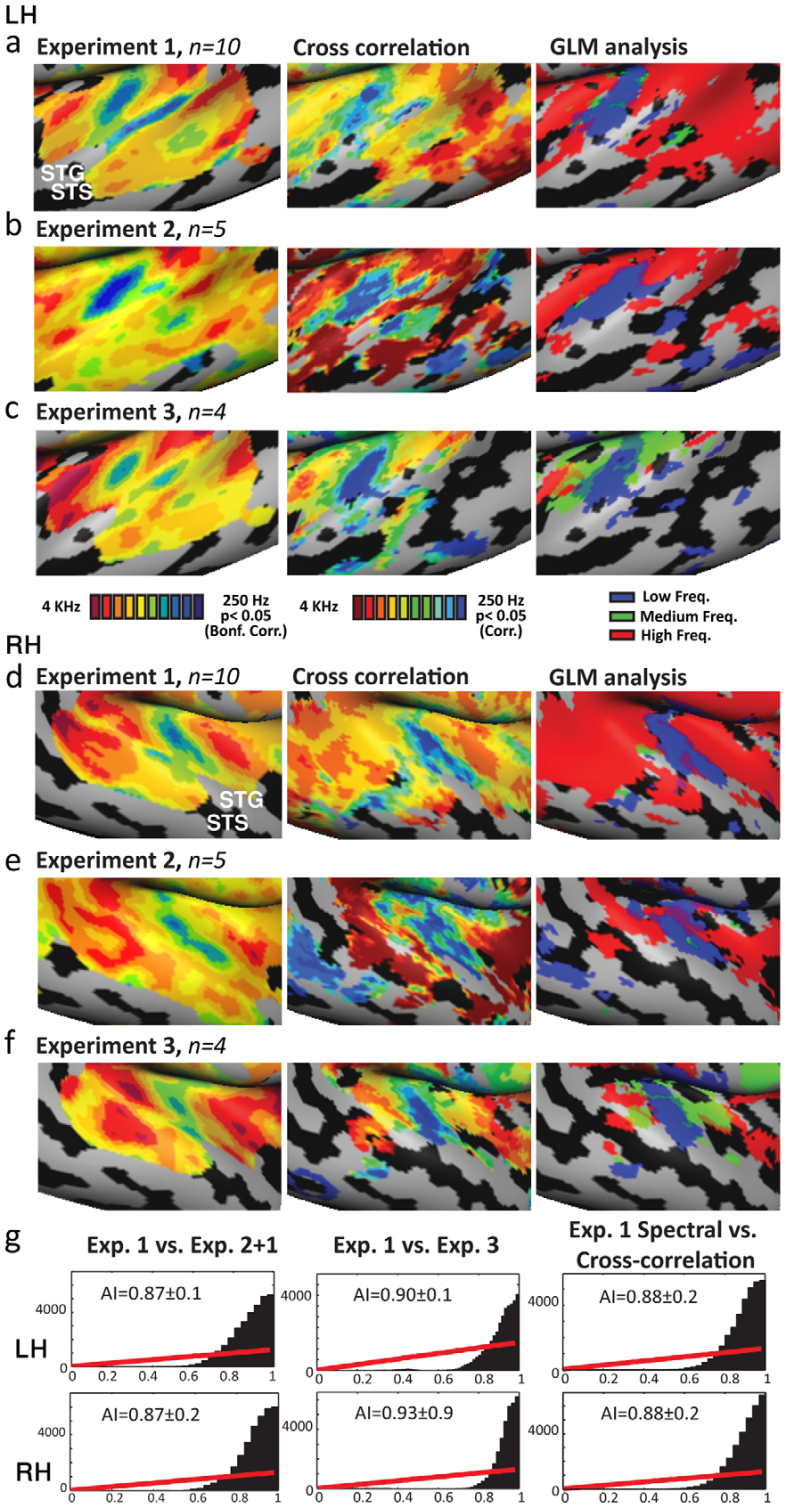
Consistency of the mirror-symmetric cochleotopic maps across experiments and analyses. **A.** (left hemisphere) and **D.** (right hemisphere) display, on the left column, auditory cortex relative frequency preference map magnification as seen in **Figs. 2** (LH) **and 3** (RH), showing the mirror- symmetric cochleotopic maps inspected using spectral analysis. In the middle column cross-correlation analysis for the averaged single-subject time-course is displayed, showing remarkably similar trends to that of the spectral analysis. On the right column, the continuous auditory stimulation was analyzed by dividing it in a random effect general linear model (RFX-GLM) into low, medium and high frequency tone conditions. The GLM map displays the contrast of each frequency band with the other conditions. **B.** (left hemisphere) and **E.** (right hemisphere) display cross-correlation (middle) and GLM (right) analyses for Exp. 2 (*n = 5*) in which the chirp was reversed (i.e. from high to low frequencies). Spectral analysis (left) is the averaged phase map of Exp. 2 with Exp. 1, thus fully controlling for the hemodynamic delay of both experiments ([52], [53]; for the spectral maps of Exp. 2 alone see **Fig. S5**). The consistency of these results with the main experiment show that the auditory fields and cochleotopic gradients displayed for Exp. 1 do not result from the frequency modulation direction. **C.** (left hemisphere) and **F.** (right hemisphere) display spectral (left), cross-correlation (middle) and GLM (right) analyses for Exp. 3, in which a subgroup (*n = 4*) of subjects was scanned again one month after the original scan, revealing similar patterns of iso-frequency bands as the original (first scan) map, demonstrating the high test-retest reliability of the auditory fields and their locations. See also **Fig. S8** for further single subject analysis of this experiment. **G.** Similarity alignment histograms are presented for three main contrasts, between the main experiment (Exp. 1) and the two control experiments (Exps. 2 and 3) and between the spectral and cross-correlation analyses in Experiment 1, for both hemispheres. The distribution of each comparison's alignment indexes (between 0 and 1 in each comparison) show a sharp peak towards 1, demonstrating their significance, and differ significantly (p<0.00001 in all comparisons presented) from random maps indexes (marked on the histograms with red line for comparison). Therefore, the similarity indexes of the correspondence between the relative frequency preference maps across analyses and experiments support the high replicability of the cochleotopic maps.

The direction of the frequency modulation could cause attention biases towards the stimulus onset or offset or other order effects and may also cause a percept of moving or looming objects [68]. Could some of the maps that appear to be cochleotopic actually result from stimuli order or inferred spatial information? To account for this possible confound, a subgroup of five subjects was scanned again in a control experiment comprised of a falling-tone chirp (Exp. 2). The group map of this control experiment is highly consistent with that of the experiment 1, both in single subjects and group level analysis (**Figs. S5, S6**), suggesting these confounds are unlikely. Additionally, following the classical retinotopic studies of Engel and Sereno [52], [53], we averaged the phase maps of the two opposing chirp directions (Exp. 1 – rising chirp and Exp. 2 – falling chirp) to control for the hemodynamic response delay and any other possible confounds resulting from stimulus direction (**Fig. 4B,E**, *n = 5*), replicating the main findings. Spectral map averaging was also performed for the entire group of subjects (while taking into account the individual variability of response onset), showing the same consistent cochleotopic gradients. These results are presented on a corresponding Talairach normalized brain of Brodmann areas (**Fig. S7**; [****69]), confirming that the cochleotopic gradients presented here exceeded Brodmann areas 41 (primary auditory cortex) and 42, covered a substantial part of Brodmann area 22 (which corresponds in part to the auditory parabelt area [70]) and continued as far as Brodmann area 21, extending far beyond the known cochleotopic areas.

In order to verify this consistency quantitatively, the spectral maps of the rising-chirp group (Exp. 1) was tested for replicability with the falling-chirp group (Exp. 2; **Fig. S5**) and with the averaged results from Experiments 1 and 2 (**Fig. 4G**). Alignment indexes between the experiments were highly significant (**Fig. 4G**: between Exps. 1 and 2: right hemisphere 0.91±0.1, p<0.00001, left hemisphere 0.9±0.09, p<0.00001, between Exp.1 and the averaged Exps.1+2: right hemisphere 0.87±0.2, p<0.00001, left hemisphere 0.87±0.09, p<0.00001). Maps' similarity can also be implied from the distribution of the alignment indexes (marked in black in **Fig.4G**). A peak towards index value of 1 implies high similarity between the maps, compared with linearly increase of random distribution of alignment indexes (in red line).

Moreover, to further validate the reliability of our results across scans, four of the ten subjects were scanned again (Exp. 3), a month following the first scan. The returning subject group results are also highly consistent with the maps from the original group (**Fig. 4C, F**, for single subject analysis see **Figs. S6, S8**). Alignment indexes of the test-retest comparison (**Fig. 4G**) were highly significant (right hemisphere 0.93±0.9, p<0.00001, left hemisphere 0.9±0.08, p<0.00001).

**Figure 5 pone-0017832-g005:**
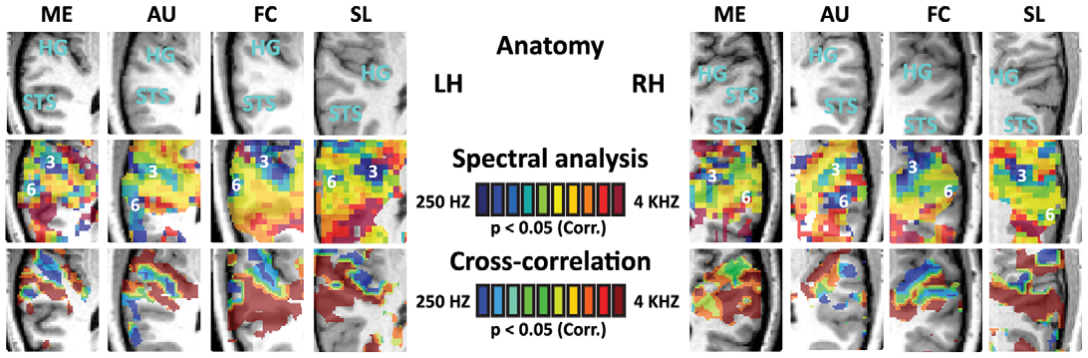
Multiple cochleotopic maps in single subjects. Anatomical structures of the horizontal views of each subject in the magnified area of the auditory cortex, unsmoothed spectral analysis relative frequency preference maps (individual R>0.18, df = 299, p<0.05, corrected for multiple comparisons) and cross-correlation maps (p<0.05, corrected for multiple comparisons) and shown for four different subjects (these and similar maps from additional horizontal slices are presented in **Fig. S6**). Overlaid on the spectral analysis maps are numbers (3, 6) representing the low-frequency peaks corresponding to those presented in the group results. Point 3 corresponds to the border between A1 and R and point 6 represents the low frequency band on the lateral STG possibly corresponding to a homologue of area CL of the belt. Single subject maps show cochleotopic maps that extend beyond the auditory core to the superior temporal gyrus and superior temporal sulcus. HG - Heschl's gyrus, STS – Superior temporal sulcus.

As the relative frequency preference maps of individual subjects (**Figs. S3, S4**) suggest, the pattern of these novel iso-frequency bands was also consistent across subjects for both hemispheres. The similarity of cochleotopic maps between subjects was tested with a pair wise alignment index (see methods). Each of the alignment indexes was found significantly different from random (p>0.0001, Bonferroni corrected for multiple comparisons), with average value of 0.81±0.075. The putative borders of all the new cochleotopic maps in the group (white lines in **Figs. 2** **,** **3** left upper panel) are presented on the individual subjects' maps (**Figs. S3B, S4B**), further demonstrating the high similarity in the location and number of the maps at the single subject level. An additional view of the cochleotopic maps of four individual subjects on their anatomical recordings (**Fig. 5**, also see for more views in **Fig. S6**) also demonstrates the cochleotopic maps in extra-core temporal cortex, extending to the superior temporal gyrus and parts of the superior temporal sulcus.

To verify the results of the phase analysis (**Figs. 1E** **,** **2** **,** **3**) through complementary analyses methods, we conducted standard cross-correlation (an alternative method used for retinotopic mapping: e.g. [71], [72]) and general linear model (GLM) analyses. Cross-correlation was used to compute the lags (in TR resolution units i.e. 1.5 sec) within a stimulus cycle at which each voxel correlated best to the frequency of stimulation (i.e. in cochleotopic mapping, its preferred tone). Cross-correlation maps of the averaged single-subject data was highly consistent with the spectral analysis maps, in all three experiments (**Fig. 4**, middle column), and proved consistency in single subject level (**Fig. 5**, extended at **Fig. S6**). The similarity of the cross-correlation analysis and the phase analysis (**Fig. 4G**) results confirms that the two analyses yielded statistically similar cochleotopic gradients (alignment index of 0.88±0.16 for both hemispheres, p<0.00001). GLM analysis was also used to independently assess the preferred tone of each voxel (though it is less optimal for the current design and generally less sensitive for topographical mapping, e.g. it is rarely used in retinotopic experiments). To compute this, the continuous auditory stimulation was divided into three separate equal intervals: low, medium and high frequencies. In each experiment, the activation elicited by each of the three intervals was contrasted to that of the other intervals, resulting in random effect GLM contrast maps (e.g. high vs. medium and low; etc. **Fig. 4**, right column). While these complementary analyses are statistically weaker than the main spectral analysis technique, the same trends can be seen across analyses.

**Figure 6:**
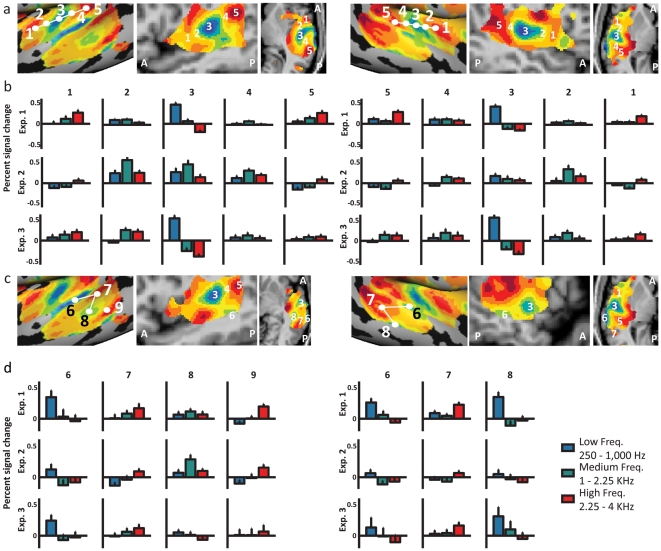
Cochleotopic maps in the human auditory cortex verified by RFX-GLM raw time-course analysis. **A.** Auditory cortex relative frequency preference map magnification is the same as in **Figs. 2** **and** **3**, with points (1–5, see Talairach coordinates at **Table 1**) along the auditory core gradients that were used to sample individual time-courses and compute random effect GLM time-course analysis. The approximate location of the same sampling points is also presented in a volume view of sagittal and horizontal slices. **B.** Time-courses of activation and response averages were sampled from points (1–5) along the anterior-posterior cochleotopic gradient (of the core areas), in **both** cortical hemispheres. Response averages were calculated for Exp. 1 group (*n = 10*), Exp. 2 group (falling chirp, *n = 5*) and for Exp. 3 group (scan repetition, *n = 4*) from the same locations. The continuous auditory stimulation was analyzed by dividing it in a random effect general linear model (RFX-GLM) into low, medium and high frequency tone conditions. Error bars denote standard error of the mean (SEM). Tone preference examined using this complementary analysis was consistent with relative frequency preference maps revealed by spectral analysis. **C.** Auditory cortex relative frequency preference map magnification is the same as in **Figs. 2** **and** **3**, with points (6–9) marking the lowest and highest frequency tones which represent the mirror-symmetry flipping points between the extra-core cochleotopic maps (see Talairach coordinates at **Table 1**). These points were used to sample individual time-courses and compute random effect GLM time-course analysis, similarly to A-B. The approximate location of the same sampling points is also presented in a volume view of sagittal and horizontal slices, with reference to the sampling points of the core. **D.** Similar to B, response averages of activation were sampled from points 6–9 in the left hemisphere, and 6–8 in the right hemisphere, along the superior-inferior cochleotopic gradient in both scan sessions, validating the tone preference of the iso-frequency bands in the extra-core areas of auditory cortex.

Additionally, we investigated the magnitude of activation assessed by the average percent BOLD signal change of the *raw time-courses of individual subjects* using random-effect GLM (**Fig. 6**). The raw time-course was sampled across the subjects from 5 points representing the mirror-symmetry flip axes of 3 iso-frequency bands seen in core areas, as well as two intermediary points (**Fig. 6B**, sampling points marked in **Fig. 6A** on the cortical view, with approximate marking of the same points on volume views), and from 3 additional points from the putative superior-to-inferior iso-frequency bands in the extra-core areas (**Fig. 6C,D** **)**, and the average magnitude of activation for each frequency band was computed. The average activation for each point was highly consistent with the tone preference presented in the phase maps, thus confirming the reliability of our phase analysis in determining tone selectivity of both core and novel extra-core cochleotopic regions. Furthermore, the average response patterns were replicated in individual subjects (see averaged responses in **Figs. S3B, S4B**) and additionally was replicated across experiments in the falling-chirp experiment (Exp. 2, *n = 5*; **Fig. 6B,D**) and across scanning days on the second scan (Exp. 3, *n = 4*; **Fig. 6B,D**) using the same sampling points selected in the main experiment (Exp. 1), demonstrating the reliability of the cochleotopic maps reported here across subjects and scan days.

## Discussion

Using spectral analysis fMRI, we showed that: 1. There may be as many as 6 cochleotopic maps in the human cerebral cortex: at least two core areas, corresponding to A1 and R (and their neighboring belt areas), and possibly RT, (see **Figs. 2** **,** **3** **, Fig. S2**) and as many as 4 novel cochleotopic maps in the temporal cortex (see **Figs. 2** **,** **3** **,** **4** **,** **6**, **Figs. S3, S4** and **Movie S1**). 2. Cochleotopic preference is by no means limited to the auditory core but rather extends to the higher-order auditory regions within the temporal lobe (as far as the STS/MTG, see **Figs. 2** **,** **3**, **Figs. S3, S4, S7** and **Movie S1**). 3. Cochleotopic maps in high-order auditory areas are also arranged in a mirror-symmetry organization (see the borders of mirror symmetry drawn in **Figs. 2** **,** **3**, **Figs. S3, S4** and **Movie S1**), which may help define and parcel the auditory cortex into distinct auditory fields (for example, as done in **Figs. 2** **,** **3**, left upper panel). 4. It would appear that similar to the visual cortex, the auditory cortex (at least in the temporal lobe) is also fundamentally topographical in nature, which may suggest that this large-scale governing principle of organization is sensory modality invariant.

Previous work has provided evidence for the existence of cochleotopic mapping in core areas, probably the human homologues of areas A1 and R [35], [38], [41], as well as a thorough cochleotopic mapping of the surrounding belt areas in primates [16], [17], [18], [19], [21], [30], including using fMRI in a high-field 7T fMRI scanner [30]. Two studies [37], [41] examined the cochleotopic mapping in humans along the superior temporal gyrus and showed that it may also extend, as is seen in primates, to the areas immediately surrounding the human auditory core, which may correspond to some of the belt areas. However, our study is the first to look for the cochleotopic mapping of the entire human cortex, a mapping which was enabled by the combination of a continuous chirp stimulus, spectral analysis (which reveals also widely-tuned responsive regions), and whole brain scanning. In addition to providing data from single subjects using unsmoothed data, our relatively large group of subjects allowed us to develop and apply group analysis that enabled us to generalize spectral analysis in cochleotopy to the population level [65], which allows to look at similarities between single subjects analysis not only by comparing individual maps (as also done here in **Fig. 5** **, Figs. S2, S6, S8**, and by applying a similarity measure of alignment index) but also to look for consistent large-scale results in the group level (for review on the importance of this approach combined with single subject analysis see [65]). Here we demonstrate that even the higher-order human auditory cortex, outside the traditional cochleotopic regions, is organized on a large scale by cochleotopic gradient patterns. This analysis method allowed us to delineate the location and frequency gradients in uncharted cortical cochleotopic regions, which we interpret as distinct auditory cortical fields (which may, of course, be further divided according to functional or anatomical markers, for example the division of caudal and rostral parabelt fields [15]). These cortical fields are organized in a mirror- symmetric fashion, and are consistent across subjects and across recording days, suggesting that they correspond to segregated anatomical areas employed in auditory processing. There appear to be two mirror-symmetry axes: one centered around Heschl's gyrus in the anterior-posterior axis (**Figs. 2** **–** [Fig pone-0017832-g003] [Fig pone-0017832-g004] [Fig pone-0017832-g005] **6**), which is likely to correspond to the core and belt areas, as seen in primates and humans [30], [41] and the other one is stretching from STG to STS (**Figs. 2** **–** [Fig pone-0017832-g003] [Fig pone-0017832-g004] [Fig pone-0017832-g005] **6**), which may extend to the parabelt areas. The first mirror-symmetric axis is in accordance with previous literature of both humans and primate findings (and see details below), demonstrating the cochleotopic mapping in the core auditory cortex, including areas A1 and R (corresponding to gradient between 2 high-frequency bands encompassing a low-frequency band centered around Heschl's gyrus) and the surrounding belt areas, most of which continue the cochleotopic gradients of the core areas, and thus identifiable only by their cytoarchitecture [20], [22], [24], [73], [74] or functional differences (such as lower responsiveness to pure tones [34], [45], [56]; for example of functional discrimination of the core and belt using this principle see in [30]). Our results mirror the gradient direction of the core as presented in several recent findings [37], [41] who reported gradients perpendicular to the long axis of Heschl's gyrus (i.e. isofrequency bands along the HG long axis, and gradients in an anterior-posterior axis), rather than gradients following a slightly more oblique orientation, more resembling that of the anatomy of Heschl's gyrus [35]. The topic of gradient direction of the core is currently still debated in humans. Recent findings [41] comparing surface and volume cochleotopic maps suggest that this controversy may have at least partially resulted from the resolution of the cochleotopic gradients with regard to the proximity of the sulci bordering Heschl's gyrus, and their high intersubject variability. While our study does not focus on the core gradients (also due to the limited spatial resolution), it offers support to the posterior–anterior axis of core gradients, in hope that future high-resolution studies focusing on the exact anatomical-functional link in Heschl's gyrus will contribute to the resolution of this debate.

In addition to the well-known core gradients of A1 and R, the first mirror symmetric axis includes partial evidence for an additional low frequency focus anterior to the high-low-high bands (evident in the right hemisphere) on the surface of the superior temporal gyrus, possibly corresponding to area RT of the macaque [30] for which inconclusive results have been seen in previous human studies [41]. However, further high-resolution scans specifically analyzing individual subjects in this area (for instance to see if this gradient indeed exists in the temporal plane of both hemispheres) is required to reach decisive conclusions as to the existence in humans. In addition to the core gradients, a low-frequency band somewhat perpendicular to the low frequency band along the axis of HG (in which sampling point 6 was taken in **Fig. 6**) may at least partly correspond to posterior belt field CL of the macaque (whose cochleotopic gradient does not continue that of the core, and is thus easily definable, according to findings in primates and humans [30], [34], [40], [41], thus defining the possible extent of the belt to the cochleotopic areas within the superior temporal gyrus and Heschl's gyrus. This functional localization and its correspondence to the known gradients of the belt in macaques and humans enables us to assume that the additional maps in the more inferior temporal lobe, corresponding to the second mirror-symmetry axis, specifically in the superior temporal sulcus, belong to the parabelt and other high-order auditory regions, extending beyond the belt to areas corresponding to CP and RP of the parabelt [15], [21], [27], [28], [75], and perhaps beyond them. Superimposing our cochleotopic maps on the Talairach normalized Brodmann areas [69] further demonstrates that the maps within the superior temporal sulcus and inferior to it coincide with Brodmann area 22 (**Fig. S7** and **Table 1**), parts of which are considered to be homogenous to the auditory parabelt [70], to areas in which selectivity for complex auditory properties has been demonstrated, for example voice-sensitivity preference [43] and preference for animals vs. tools sounds [44].

**Table 1:** Talairach coordinates of mirror-symmetry flipping points between the cochleotopic maps in the temporal lobe.

Sampling point	X	Y	Z	Brodmann Area
LH				
1	−43	−16	2	BA 22
2	−46	−16	10	BA 41
3	−45	−22	10	BA 41
4	−48	−28	14	BA 41
5	−55	−35	20	BA 42
6	−64	−23	7	BA 42
7	−54	−36	9	BA 22
8	−42	−30	3	BA 22
9	−56	−37	5	BA 22
RH				
1	38	−19	1	BA 22
2	40	−18	8	BA 22
3	46	−21	9	BA 41
4	50	−24	10	BA 41
5	54	−30	16	BA 42
6	62	−22	7	BA 42
7	47	−29	5	BA 22
8	63	−27	5	BA 22

Although it is extremely difficult to link a cochleotopic gradient observed at a greater neuroimaging resolution in a human group to a specific cytorachitectonically, anatomically defined region (especially due to the high inter-subject variability of such cytoarchitecture structures, as well as discrepancies between different anatomical methods [20]), we will attempt to suggest a putative initial model of how our observed cochleotopic gradients correspond to the extensive anatomical mapping effort of the human auditory cortex, according to the most recent and elaborate architectonic scheme suggested by Fullerton and Pandya [20]. According to their anatomical division of the human auditory cortex into multiple fields, our functional cochleotopic division is likely to refer to (and extend beyond) a large number of cytoarchitectonically-defined fields, within the core (divided into as many as 8 fields; Ts1I, Ts2i, PaAr, Km(1–3), Kl, PaAc [20]), lateral belt (divided into as many as 6 fields; Ts1e, Ts2e, Ts3, PaAi, PaAe, Tpt) and medial belt or root (divided into as many as 4 fields; ProI, PaI, ProK, Reit) auditory cortex. The multiplicity of the anatomical fields with regard to cochleotopic gradients suggests that the link between anatomy and topographic gradients is a complex one. While the cytoarchitectural fields may indeed represent segregated functional units comparable to the elaborate auditory functions in the human brain, they may still share cochleotopic gradients, as is indeed known for the continuous gradients between the core and some of the belt areas in the macaque [30]. For example, areas KI and Km1 to Km3 of the core and areas ProI, PaI, ProK of the root (or medial belt) appear to be organized in parallel and in a general perpendicular angle to the long axis of Heschl's gyrus [20], along the anterior-posterior cochleotopic gradient of this part of the core and belt (corresponding to our sample points 1–5 in **Fig. 6**). When comparing human cytoarchitectonic mapping [20] and macaque [30] and human [35], [37] cochleotopic mapping, the border line between KI+Km1 and Km2+3 in the core may correspond to the border between A1 and R, thus forming the mirror-symmetric axis of the core (on which sampling point 3 in **Fig. 6** is located), which is continued to belt area CM of the macaque, possibly corresponding to cytoarchitectural areas PaAc and Reit in the belt and root [20]. This gradient-axis also continues anteriorly, or rostrally, to the root areas ProI, PaI, ProK, which may be located in the high-frequency selective area at the anterior end of this gradient. The additional gradient we assume corresponds to area CL of the macaque (sampling point 6 in **Fig. 6**), manifesting most prominently in an additional low-frequency selective band, may correlate to the approximate location of areas PaAe, PaAi and possibly bordering Tpt [20]. It is more difficult to determine how the additional superior-inferior cochleotopic gradients (sampling points 7–9 in **Fig. 6**) correspond to cytorachitectonic structures. There is already inconsistency in the interpretation of different studies to the exact architectonic organization of these more lateral areas (for example areas PaAi and PaAe), and to their definition as belt [12], [20]] or parabelt [15], [25], [75] auditory cortex. In any case, due to the consistent mapping of all cytoarchitectural core, root and belt areas to the superior temporal plane extending maximally to encompass STG, and the functional mapping of the corresponding areas to cochleotopic mapping on the anterior-posterior axis [30], we can assume that the additional cochleotopic gradients we observed (sampling points 7–9 in **Fig. 6**) extend beyond the core, root and belt at least to the (less charted and agreed upon) parabelt fields and perhaps beyond them, to anatomical areas numbered by Brodmann [69] as parts of areas 22 and 21, (as can also be seen in **Fig. S7**, presenting our cochleotopic mapping on an anatomical estimate of the Brodmann areas). Ultimately, the attribution of the in-vivo cochleotopic functional data to the detailed anatomical division of the human auditory cortex can best be accomplished by future studies applying high-resolution microanatomy MRI analysis in addition to cochleotopic mapping to identify individual lamination structures and their functions (for example, [76] applying such analysis to the visual cortex).

Here we found multiple cochleotopic maps in the temporal lobe, however we did not find consistent maps beyond it. Interestingly we did find several other regions which showed significant responses to our stimulation protocol (i.e. highly significant correlation coefficient in all three experiments; see **Fig. S1**). These regions included bilateral activation in the posterior-inferior frontal lobe, medial superior frontal gyrus\ premotor cortex, precuneus, and a left inferior parietal cluster, regions sporadically reported previously to be involved in various auditory localization and recognition tasks [48], [50], [77], [78]. However none of these regions showed clear and consistent cochleotopic arrangement across the experiments and between the subjects. This does not preclude finding such maps in the future, using for instance specific stimuli that match the functional preferences of these areas, higher resolution and focused scanning of such specific areas outside the temporal cortex. Additionally, we did not observe a clear cochleotopic gradient, but rather a general preference for medium and high-frequency tones, caudally to the core and belt in the planum temporale (PT; part of which corresponds to Tpt of the monkey and possibly of the human [20], [23], [25], [79]). This area is thought to be involved in speech processing [80], [81], perhaps as part of a computational hub for discerning complex spectrotemporal auditory objects and their locations [82], [83], and may thus have been less likely to be activated by our relatively simple stimulus type (i.e. tonal sweep). Future studies directing their attention to more complex and appropriate stimuli may reveal this the tonal preference of this area.

Our results clearly verify the organization of core areas [35], [38] previously reported. However they also show that at least two large-scale previously unreported cochleotopic maps beyond core and belt areas exist in the right hemisphere, and at least three exist in the left hemisphere (with mirror symmetry axis flipping from STG to STS (**Figs. 2** **,** **3**). This is a conservative estimate, and there are hints that additional maps may be present in the occipito-temporal cortex. These areas clearly extend far beyond typical core [35], [38] and belt [37], [41] areas, all the way to superior temporal sulcus, well into regions which are traditionally considered non-cochleotopic [56], and which engage both in complex auditory processing and multisensory integration [43], [44], [45], [84], [85], [86], [87]. Our results show that while even relatively early auditory cortical areas may exhibit sensitivity to complex sound features [46], [87], the large scale organization of most of the auditory cortex still maintains clear cochleotopic preferences. Combining our results with previous studies, suggests that the organization of the auditory cortex may be that the cochleotopic mapping is relatively coarse, stretching all the way from A1 to STS (covering core, belt and parabelt areas) while local populations of neurons are more heterogeneous in their preference. Indeed recent studies found [88], [89] that the cochleotopic mapping in primary auditory cortex of mice is only present on a (relatively) large scale, whereas local neuronal populations show less organized cochleotopic gradients. It will be interesting to combine in future studies between cochleotopic mapping and high order auditory functions (like voice recognition). For comparison's sake, it is useful to look at the balance between topographical mapping and functional specialization in the visual modality. It is important to note that visual fields may have combined eccentricity, polar angle and other specific functional characteristics. For example, V4 shows color sensitivity in addition to being part of a large scale retinotopic organization; the fusiform face area (FFA) has a combined preference for faces and a foveal retinotopic eccentricity preference, and the parahippocampal place area (PPA) has combined selectivity for place stimuli and a peripheral retinotopic eccentricity preference [2], [90]. In the same manner, a given auditory area might have several orthogonal receptive field characteristics (for example preference for species-specific vocalizations or communication calls [43], [45]); one of them appears to be, as our findings show, a cochleotopic topographical organization.

Along this line, multiple topographical maps delineating parallel and divergent functional regions have been accounted for in terms of computational efficiency or even developmental and evolutionary advantages and efficiency [91], [92]. As found in the visual cortex, which is mapped both according to polar and eccentricity topographical mapping, additional axes of topographical mappings may be exposed in the auditory cortex in the future, possibly, for instance, in modules for tuning width or binaural interactions [29], [55]. A recent study [93] suggest that the additional axis may be that of temporal sound features, with slower temporal modulation rates represented more medially and faster modulation rates more laterally on Heschl's gyrus, creating an additional modulation axis orthogonal to cochleotopic gradient, at least in the core areas. Such additional axes may, in turn, aid in better defining the axes of frequency selectivity shift, as was done in the visual domain [53]. Our results, in addition to the well-known retinotopic mapping of the visual cortex (including that of high-order visual cortex, and parietal and frontal spatial maps [2], [3], [4], may indicate that topographical mapping could be a more common, fundamental principle of sensory cortex organization well beyond primary and secondary cortices, perhaps as suggested previously [91], [92], due to its computational advantages.

The discovery of new cochleotopic map borders might greatly aid future characterization of the functions of the new auditory fields in humans, and help define their possible connectivity patterns, interactions and hierarchical processing structure, as well as test them with regard to the suggested theoretical framework of the two auditory processing streams [7], [26], [48], [49], [50], [51]. For example future studies in ultra-high field scanners further exemplifying cochleotopic mapping in the prefrontal lobe may be able to show cochleotopic mapping in the prefrontal cortex, and even draw a cochleotopic border between the ventrolateral (VLPFC) and dorsolateral (DLPFC) prefrontal cortex, thought to be high-level stations in the dual ‘what’ and ‘where’ processing scheme [49], [50], thus testing in more detail this hypothetical model. It will also allow for better comparisons between results reported in different studies and standardize references to them (as opposed to comparisons on the basis of brain anatomy such as gyri and sulci which are very variable with relation to function [67]), which will also potentially lead to better theoretical comprehension of their common characteristics across studies.

The understanding of the topographical structure of the auditory cortex could also be utilized for studying plastic changes in the auditory cortex in a non-invasive manner: both changes which are specific to the tonal frequency, for instance in musicians [94], or to a specific auditory function (and perhaps auditory stream), such as auditory localization in the blind [95]. Additional possible future directions are the study of the plastic changes of the cochleotopic maps in congenitally blind individuals [96], in general (due to their excessive reliance on non-visual senses) and also following the prolonged use of auditory sensory substitution devices [97]. Other fascinating lines of research are the effect of tinnitus on auditory fields [98], and monitoring the awakening of auditory cortex cochleotopic responses following cochlear implants at different ages and various types of deafness [99]. Such studies may provide opportunities to study the development, plasticity and other characteristics of cochleotopic maps, an opportunity with no parallel to date in retinotopic mapping and vision.

### Conclusions

Using spectral analysis fMRI, we showed additional cochleotopic maps in the human temporal lobe outside the auditory core and belt. Cochleotopic preference is thus by no means limited to the auditory core or belt but rather extends to the higher-order auditory regions within the temporal lobe, as far as the multisensory cortex in STS/MTG, extending at least to auditory parabelt areas. Cochleotopic maps in high-order auditory areas are also arranged in a mirror-symmetry organization, which may help define and parcel the auditory cortex into distinct auditory fields. It would appear that similar to the visual cortex, the auditory cortex (at least in the temporal lobe) is also fundamentally topographical in nature, which may suggest that this large-scale governing principle of organization is sensory modality invariant.

## Supporting Information

Figure S1 **Auditory-responsive areas outside the temporal lobe.** Conjunction of significant (p<0.05, Bonf. Corrected) correlation coefficient maps of all 3 experiments is presented in medial and lateral views of the inflated cortical hemispheres of the standard MNI brain transformed to Talairach coordinates. In addition to the auditory-responsive areas within the temporal lobe, several regions showed significant auditory response patterns at the group level. These regions included bilateral activation in the posterior-inferior frontal lobe, medial superior frontal gyrus\ premotor cortex, precuneus, and a left inferior parietal cluster. While these areas showed correlation to the auditory stimulus timing, they did not present a clear and consistent cochleotopic arrangement. CS – Central sulcus, IFS – Inferior frontal sulcus, STS – Superior temporal sulcus.
(TIF)

Figure S2 **Single subject cochleotopic maps of the auditory core.** A. Relative frequency preference maps are shown for each of the ten subjects. A horizontal view of each subject's brain is shown, with the delineated (cyan lines) borders of Heschl's gyrus (HG). Unsmoothed relative frequency preference maps are shown in individual highly significant responsive auditory areas (highly significant Pearson's R of the correlation between the time-course and the pure cosine model, R>0.26, df = 299, p<0.05, corrected for multiple comparisons). Single subject maps display a gradual cochleotopic preference shift in their native, unsmoothed, resolution. The maps demonstrate that the core auditory cortex large- scale mirror symmetric cochleotopic mapping in the human homologues of regions A1 and R is present across subjects (in 9/10 subjects). Moreover, there is evidence of a medial-lateral cochleotopic gradient on the medial part of HG in some (6/10) subjects. Additional posterior-lateral cochleotopic gradients outside the core areas can be seen in some of the subjects even in horizontal views of the brain. For a full view of the extra-core maps see **Fig. 5**, **Figs. S3, S4, S5** displayed on the cortical surface and horizontal slices, and for test-retest reliability see **Fig. S7**). On the lowest panel for each subject, Pearson's R map is displayed, with the delineated (cyan lines) borders of HG. The peak correlation in most subjects is located approximately near HG, around and posteriorly to the low-frequency peak representing the border between putative A1 and R, demonstrating a preference for simple tone stimuli and thus supporting the identification of this region as the core auditory cortex. B. Group (*n = 10*) averaged relative frequency preference map displayed on a horizontal (z = 11), a sagittal (x = 41) and a coronal (y = −16) view of a standard MNI brain, with the delineated (cyan lines) borders of HG. The maps display the cochleotopic mapping in the core and beyond it, as seen in single subjects. Note also an additional map which includes an anterior low-frequency selective area seen in the sagittal view. This cochleotopic map possibly corresponds to a human homologue of primate core area RT (Petkov *et al.*, 2006). C. Group (*n = 10*) averaged Pearson's R map displayed on a horizontal (z = 11), a sagittal (x = 41) and a coronal (y = −16) view of a standard MNI brain, with the delineated (cyan lines) borders of HG. The peak correlation is in the core auditory cortex, but is significantly high in a large portion of the temporal lobe.
(TIF)

Figure S3 **Multiple cochleotopic maps in single subjects – Left hemisphere.** **A.** Group (*n = 10*) relative frequency preference maps, as well as 3 single subjects' maps, are presented in a lateral view of the inflated left cortical hemisphere of the standard MNI brain transformed to Talairach coordinates, as displayed in **Fig. 2**. Single subjects' maps are presented, for the sake of comparison with the group results, on the standard MNI brain, in the entire significantly responsive auditory region of the group. All relative frequency preference maps are located within the groups' highly auditory-responsive region (R>0.23, P<0.05 Bonf. corr.). All maps show multiple iso-frequency bands, in addition to the known tone selectivity of the core auditory cortex. These iso-frequency bands extend in a superior-to-inferior axis along the temporal cortex. **B.** The auditory cortex region is magnified, showing the relative frequency preference map on the cortical surface. The estimated borders between the putative mirror symmetric cochleotopic maps, as acquired from the group's relative frequency preference maps (**Fig. 2**) are indicated (white line), showing the similarity of the single subject maps to the group results. Response averages of activation were sampled individually from points (1–7) along the core auditory cortex, as well as the superior-inferior cochleotopic gradient, validating the tone preference of the iso-frequency bands in the core and the accessory auditory cortex in 3 single subjects. Error bars denote standard error of the mean (SEM).
(TIF)

Figure S4 **Multiple cochleotopic maps in single subjects – Right hemisphere.** **A.** Group (*n = 10*) relative frequency preference maps, as well as 3 single subjects' maps, are presented in a lateral view of the inflated right cortical hemisphere of the standard MNI brain transformed to Talairach coordinates, as displayed in **Fig. 3**. Single subjects' maps are presented, for the sake of comparison with the group results, on the standard MNI brain, in the entire significantly responsive auditory region of the group. All relative frequency preference maps are located within the groups' high auditory-responsive region (R>0.23, P<0.05 Bonf. corr.). All maps show multiple iso-frequency bands, in addition to the known tone selectivity of the core auditory cortex. These iso-frequency bands extend in a superior-to-inferior axis along the temporal cortex. **B.** The auditory cortex region is magnified, showing the relative frequency preference map on the cortical surface. The estimated borders between the putative mirror symmetric cochleotopic maps, as acquired from the group's relative frequency preference maps (**Fig. 3**) are indicated (white line), showing the similarity of the single subject maps to the group results. Response averages of activation were sampled individually from points (1–6) along the core auditory cortex, as well as the superior-inferior cochleotopic gradient, validating the tone preference of the iso-frequency bands in the core and the accessory auditory cortex in 3 single subjects. Error bars denote standard error of the mean (SEM).
(TIF)

Figure S5 **Consistency of spectral maps across experiments.** Spectral maps are displayed for the left and right temporal lobes in all the experiments conducted in this study. Panels **A,C** and **D** replicate the spectral maps of Exp.1, averaging of Exps. 1+2 and Exp. 3 respectively, also presented in **Fig. 4**. Panel **B** shows the spectral map of Exp. 2, which is highly consistent with the main findings. For each spectral map, the alignment indices on the right indicate the quantitative similarity with the spectral map of the main study (Exp. 1; p<0.00001 for all maps).
(TIF)

Figure S6 **Multiple cochleotopic maps in single subjects.** Anatomical structures in the magnified area of the auditory cortex in horizontal views of each subject, unsmoothed spectral analysis relative frequency preference maps (individual R>0.18, df = 299, p<0.05, corrected for multiple comparisons) and cross-correlation maps (p<0.05, corrected for multiple comparisons) are shown for five different subjects. For subject ME maps of the same horizontal view are also displayed for Exp. 2 (falling chirp, lower panel) and for subject AU maps of the same horizontal view are also displayed for Exp. 3 (second scan, lower panel), showing high test-retest reliability. Single subject maps show cochleotopic maps that extend beyond the auditory core to the superior temporal gyrus and superior temporal sulcus. HG - Heschl's gyrus, STG – Superior temporal sulcus, STS – Superior temporal sulcus.
(TIF)

Figure S7 **Cochleotopic maps projected on a Talairach normalized brain of Brodmann areas.** Relative frequency preference maps of the averaged rising chirp group (n = 10) and falling chirp group (n = 5), within the groups' high auditory responsive areas (R>0.25, p<0.05 Bonf. Cor.). The map is presented on a depiction of the Brodmann's areas in a horizontal view. Brodmann areas 21, 22, 41, 42 are depicted upon the maps, and cochleotopic gradients' peaks are marked with white triangles. Cochleotopic gradients could be found beyond primary auditory areas (Brodmann areas 41,42) in the temporal lobe towards STS (Brodmann areas 21, 22).
(TIF)

Figure S8 **Single subject cochleotopic maps are consistent across repetitions.** A. A horizontal view of the auditory cortex of 4 subjects who were scanned twice in two different days (Exp. 1 and Exp. 3) is shown, with the delineated (yellow lines) borders of Heschl's gyrus. Spectral analysis relative frequency preference maps (in individual significantly responsive areas, R>0.26, df = 299, P<0.05, corrected for multiple comparisons) are shown below. Cochleotopic maps seen on the primary auditory cortex in an anterior-posterior pattern are highly replicable across scans and across subjects. B. Group averaged maps (n = 4) for the first and second scans are presented on the MNI (Montreal Neurological Institute) standard brain, transformed to Talairach coordinates. The average maps, as well as the single subject maps, are remarkably similar in the two repeated scans.
(TIF)

Movie S1 **Spread of best frequency areas according to auditory stimulus.** Group (Session 1, *n = 10*) relative frequency preference maps are presented in a lateral view of the partly inflated cortical hemispheres of the standard MNI brain, as presented in **Fig. 1E**. The video depicts the progressions of tonal frequency sensitivity in the auditory cortex. Cortical response of the group to the heard rising tone chirp is displayed in white for successive sampling points. Note the impressive mirror-symmetric pattern revealed in this tonal frequency progression movie. (can also be found at: http://brain.huji.ac.il/stuff/cochleotopy_movie.html).
(AVI)
