# Supplementary figures and images for: Extensive Cochleotopic Mapping of Human Auditory Cortical Fields Obtained with Phase-Encoding fMRI
Source: PLoS One. 2011 Mar 23;6(3):e17832. doi: 10.1371/journal.pone.0017832 (PMC3063163; doi:10.1371/journal.pone.0017832)

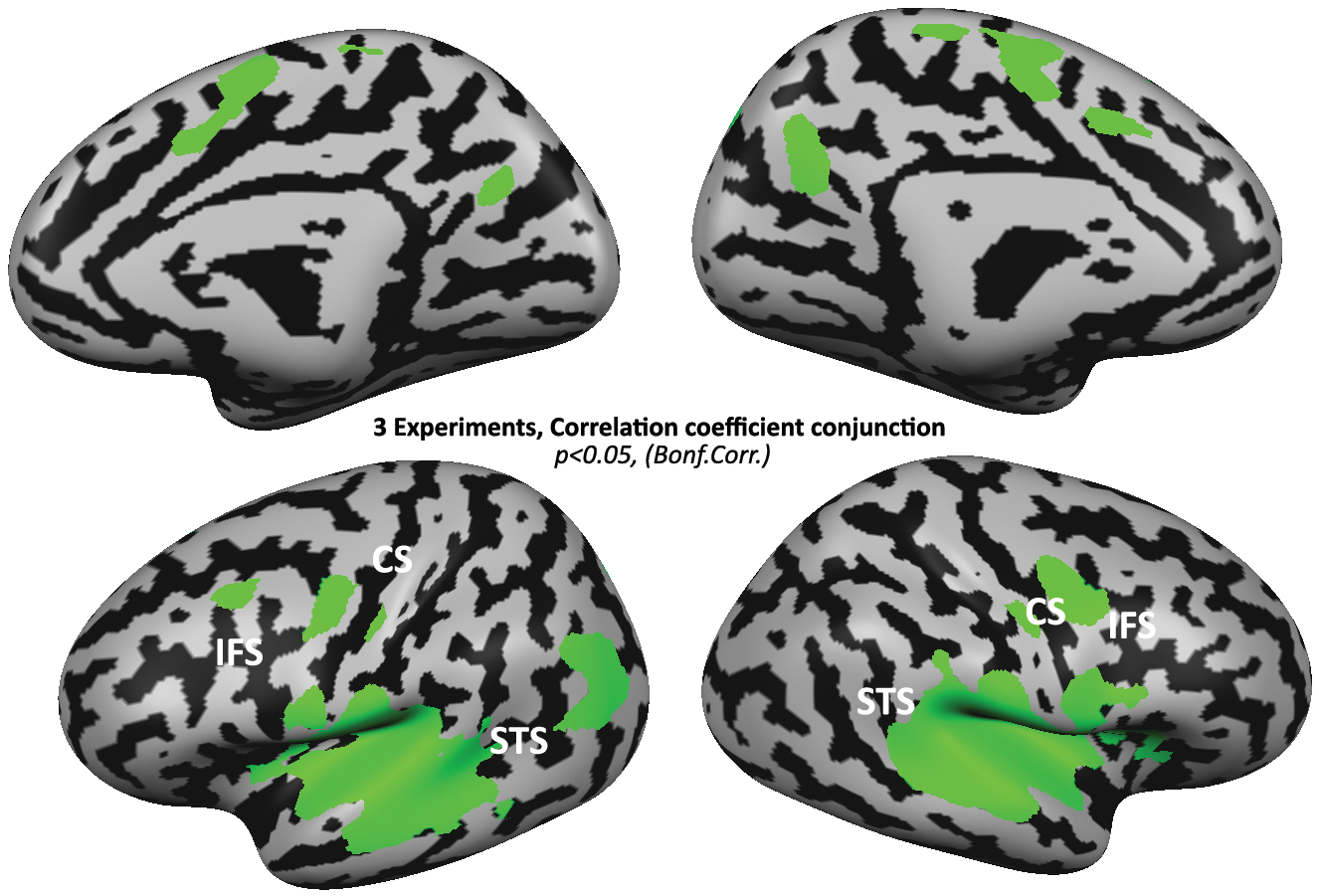

Supplement: Figure S1 — Auditory-responsive areas outside the temporal lobe. Conjunction of significant (p<0.05, Bonf. Corrected) correlation coefficient maps of all 3 experiments is presented in medial and lateral views of the inflated cortical hemispheres of the standard MNI brain transformed to Talairach coordinates. In addition to the auditory-responsive areas within the temporal lobe, several regions showed significant auditory response patterns at the group level. These regions included bilateral activation in the posterior-inferior frontal lobe, medial superior frontal gyrus\ premotor cortex, precuneus, and a left inferior parietal cluster. While these areas showed correlation to the auditory stimulus timing, they did not present a clear and consistent cochleotopic arrangement. CS – Central sulcus, IFS – Inferior frontal sulcus, STS – Superior temporal sulcus. (TIF) [file pone.0017832.s001.tif]

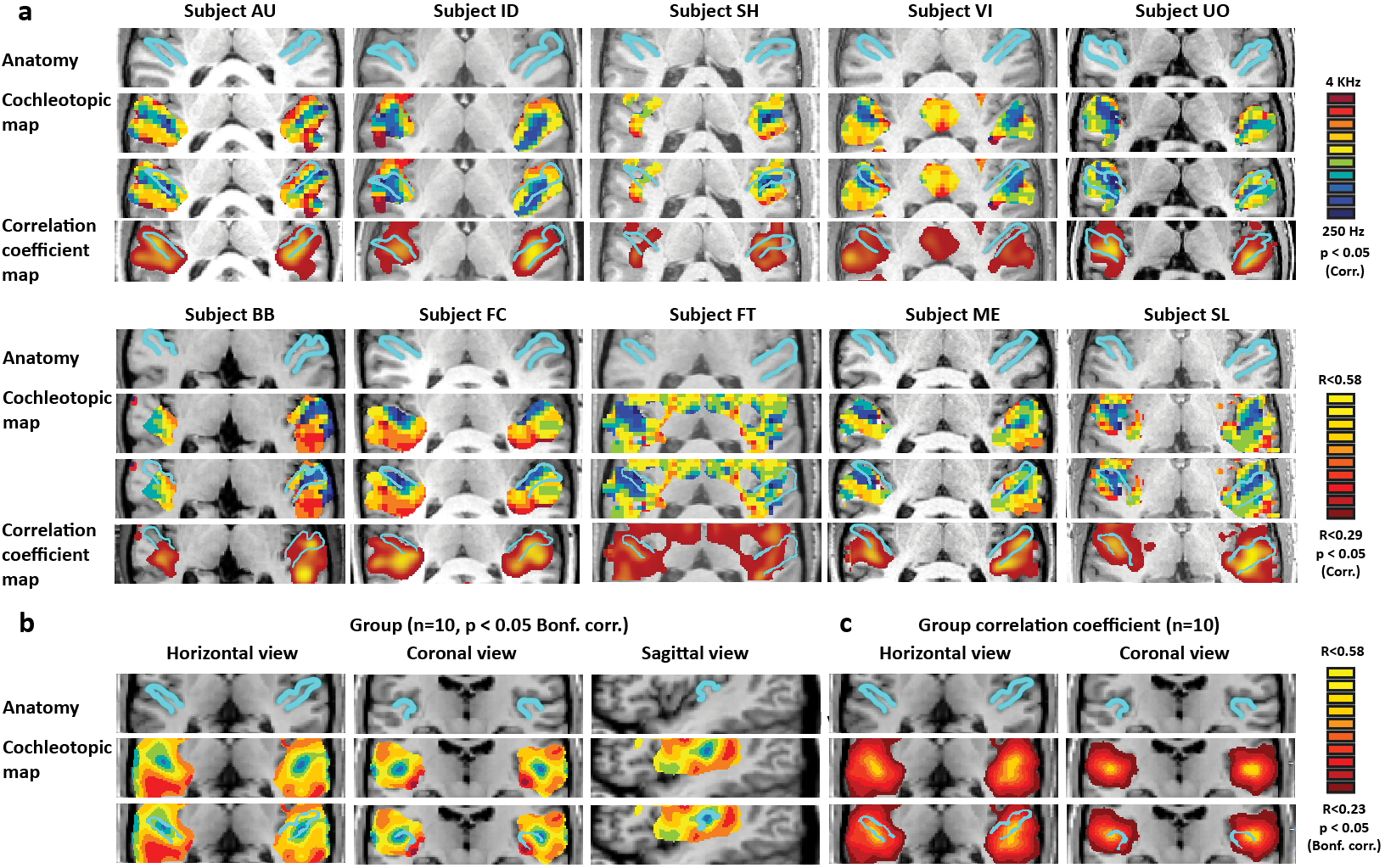

Supplement: Figure S2 — Single subject cochleotopic maps of the auditory core. A. Relative frequency preference maps are shown for each of the ten subjects. A horizontal view of each subject's brain is shown, with the delineated (cyan lines) borders of Heschl's gyrus (HG). Unsmoothed relative frequency preference maps are shown in individual highly significant responsive auditory areas (highly significant Pearson's R of the correlation between the time-course and the pure cosine model, R>0.26, df = 299, p<0.05, corrected for multiple comparisons). Single subject maps display a gradual cochleotopic preference shift in their native, unsmoothed, resolution. The maps demonstrate that the core auditory cortex large- scale mirror symmetric cochleotopic mapping in the human homologues of regions A1 and R is present across subjects (in 9/10 subjects). Moreover, there is evidence of a medial-lateral cochleotopic gradient on the medial part of HG in some (6/10) subjects. Additional posterior-lateral cochleotopic gradients outside the core areas can be seen in some of the subjects even in horizontal views of the brain. For a full view of the extra-core maps see Fig. 5 , Figs. S3, S4, S5 displayed on the cortical surface and horizontal slices, and for test-retest reliability see Fig. S7). On the lowest panel for each subject, Pearson's R map is displayed, with the delineated (cyan lines) borders of HG. The peak correlation in most subjects is located approximately near HG, around and posteriorly to the low-frequency peak representing the border between putative A1 and R, demonstrating a preference for simple tone stimuli and thus supporting the identification of this region as the core auditory cortex. B. Group (n = 10) averaged relative frequency preference map displayed on a horizontal (z = 11), a sagittal (x = 41) and a coronal (y = −16) view of a standard MNI brain, with the delineated (cyan lines) borders of HG. The maps display the cochleotopic mapping in the core and beyond it, as seen in singl [file pone.0017832.s002.tif]

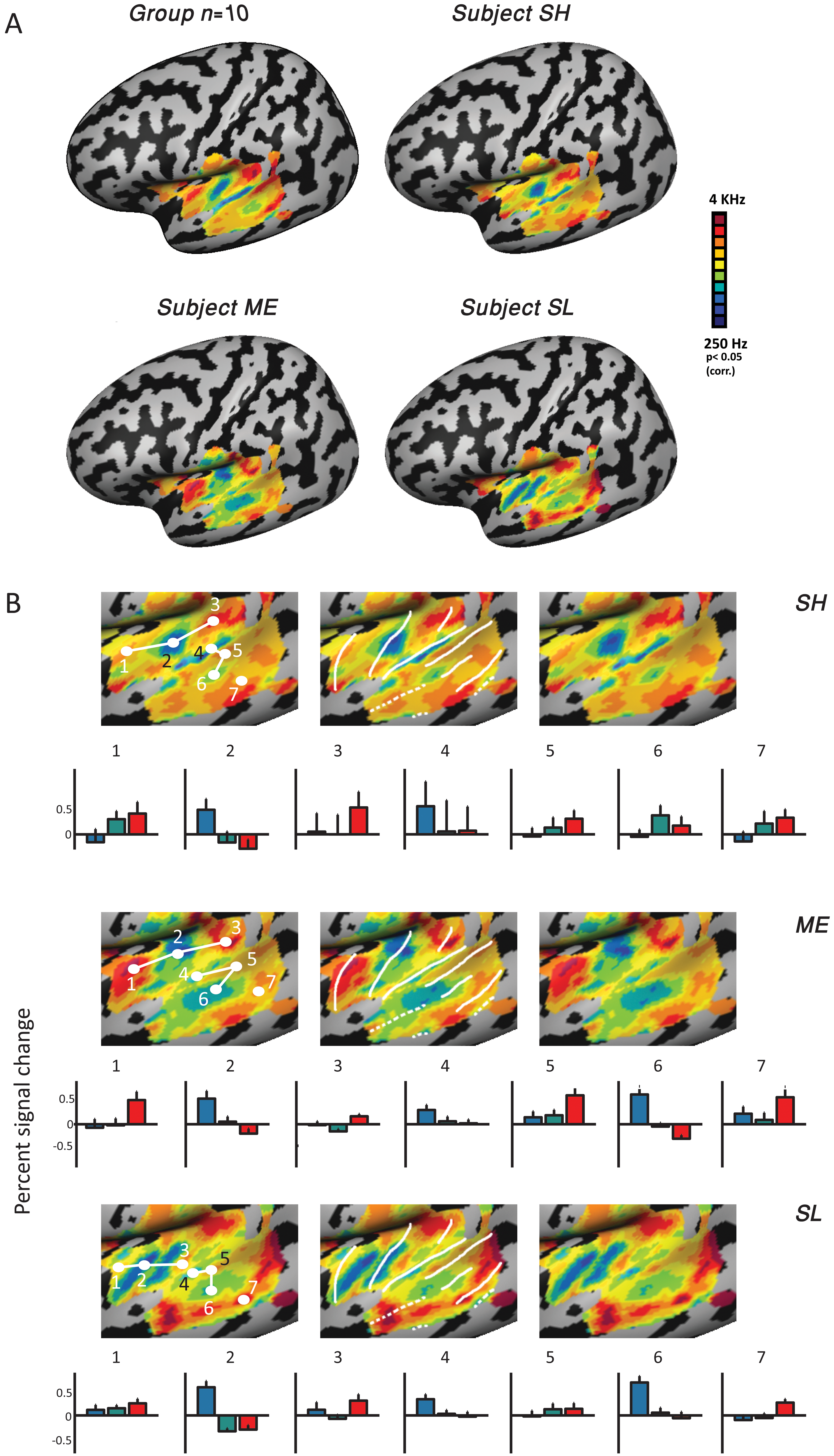

Supplement: Figure S3 — Multiple cochleotopic maps in single subjects – Left hemisphere. A. Group (n = 10) relative frequency preference maps, as well as 3 single subjects' maps, are presented in a lateral view of the inflated left cortical hemisphere of the standard MNI brain transformed to Talairach coordinates, as displayed in Fig. 2 . Single subjects' maps are presented, for the sake of comparison with the group results, on the standard MNI brain, in the entire significantly responsive auditory region of the group. All relative frequency preference maps are located within the groups' highly auditory-responsive region (R>0.23, P<0.05 Bonf. corr.). All maps show multiple iso-frequency bands, in addition to the known tone selectivity of the core auditory cortex. These iso-frequency bands extend in a superior-to-inferior axis along the temporal cortex. B. The auditory cortex region is magnified, showing the relative frequency preference map on the cortical surface. The estimated borders between the putative mirror symmetric cochleotopic maps, as acquired from the group's relative frequency preference maps ( Fig. 2 ) are indicated (white line), showing the similarity of the single subject maps to the group results. Response averages of activation were sampled individually from points (1–7) along the core auditory cortex, as well as the superior-inferior cochleotopic gradient, validating the tone preference of the iso-frequency bands in the core and the accessory auditory cortex in 3 single subjects. Error bars denote standard error of the mean (SEM). (TIF) [file pone.0017832.s003.tif]

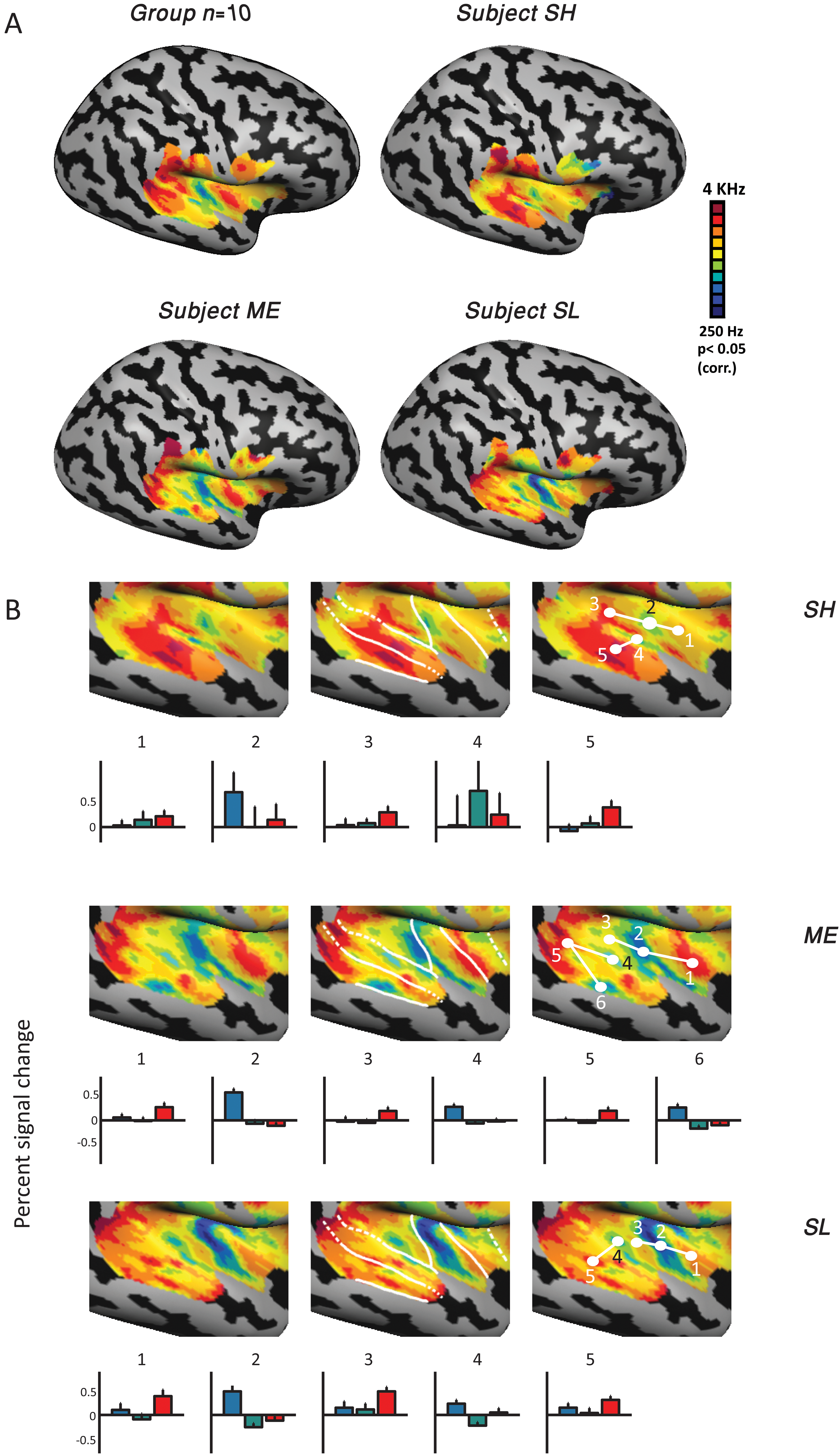

Supplement: Figure S4 — Multiple cochleotopic maps in single subjects – Right hemisphere. A. Group (n = 10) relative frequency preference maps, as well as 3 single subjects' maps, are presented in a lateral view of the inflated right cortical hemisphere of the standard MNI brain transformed to Talairach coordinates, as displayed in Fig. 3 . Single subjects' maps are presented, for the sake of comparison with the group results, on the standard MNI brain, in the entire significantly responsive auditory region of the group. All relative frequency preference maps are located within the groups' high auditory-responsive region (R>0.23, P<0.05 Bonf. corr.). All maps show multiple iso-frequency bands, in addition to the known tone selectivity of the core auditory cortex. These iso-frequency bands extend in a superior-to-inferior axis along the temporal cortex. B. The auditory cortex region is magnified, showing the relative frequency preference map on the cortical surface. The estimated borders between the putative mirror symmetric cochleotopic maps, as acquired from the group's relative frequency preference maps ( Fig. 3 ) are indicated (white line), showing the similarity of the single subject maps to the group results. Response averages of activation were sampled individually from points (1–6) along the core auditory cortex, as well as the superior-inferior cochleotopic gradient, validating the tone preference of the iso-frequency bands in the core and the accessory auditory cortex in 3 single subjects. Error bars denote standard error of the mean (SEM). (TIF) [file pone.0017832.s004.tif]

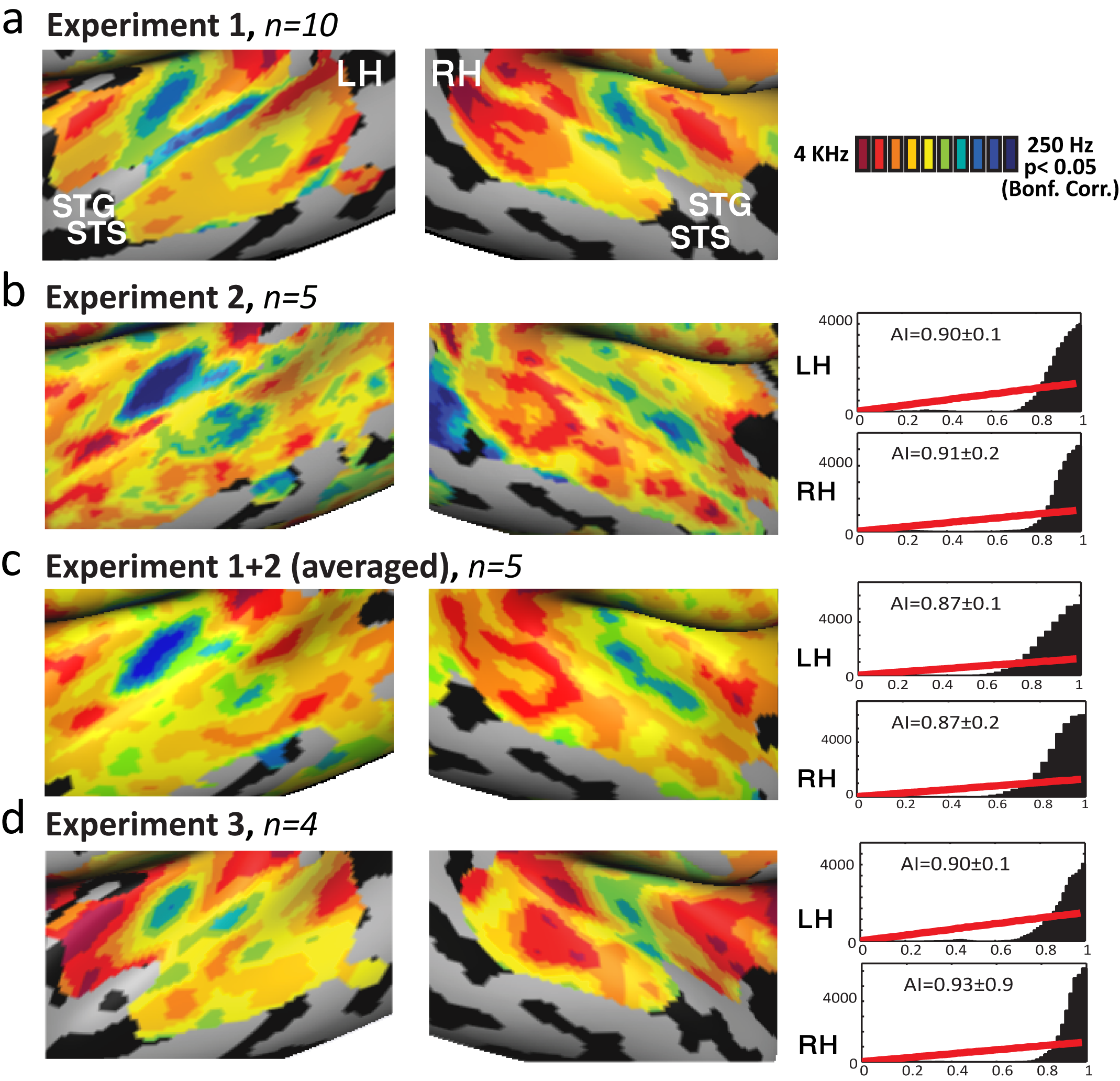

Supplement: Figure S5 — Consistency of spectral maps across experiments. Spectral maps are displayed for the left and right temporal lobes in all the experiments conducted in this study. Panels A,C and D replicate the spectral maps of Exp.1, averaging of Exps. 1+2 and Exp. 3 respectively, also presented in Fig. 4 . Panel B shows the spectral map of Exp. 2, which is highly consistent with the main findings. For each spectral map, the alignment indices on the right indicate the quantitative similarity with the spectral map of the main study (Exp. 1; p<0.00001 for all maps). (TIF) [file pone.0017832.s005.tif]

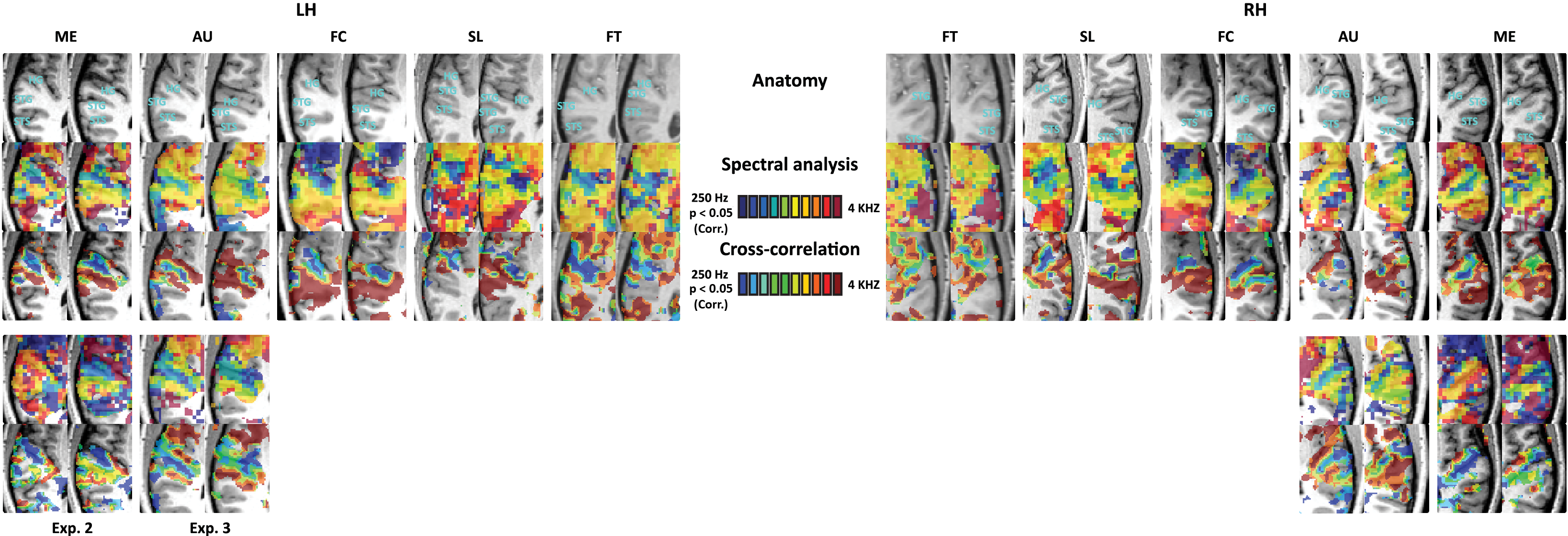

Supplement: Figure S6 — Multiple cochleotopic maps in single subjects. Anatomical structures in the magnified area of the auditory cortex in horizontal views of each subject, unsmoothed spectral analysis relative frequency preference maps (individual R>0.18, df = 299, p<0.05, corrected for multiple comparisons) and cross-correlation maps (p<0.05, corrected for multiple comparisons) are shown for five different subjects. For subject ME maps of the same horizontal view are also displayed for Exp. 2 (falling chirp, lower panel) and for subject AU maps of the same horizontal view are also displayed for Exp. 3 (second scan, lower panel), showing high test-retest reliability. Single subject maps show cochleotopic maps that extend beyond the auditory core to the superior temporal gyrus and superior temporal sulcus. HG - Heschl's gyrus, STG – Superior temporal sulcus, STS – Superior temporal sulcus. (TIF) [file pone.0017832.s006.tif]

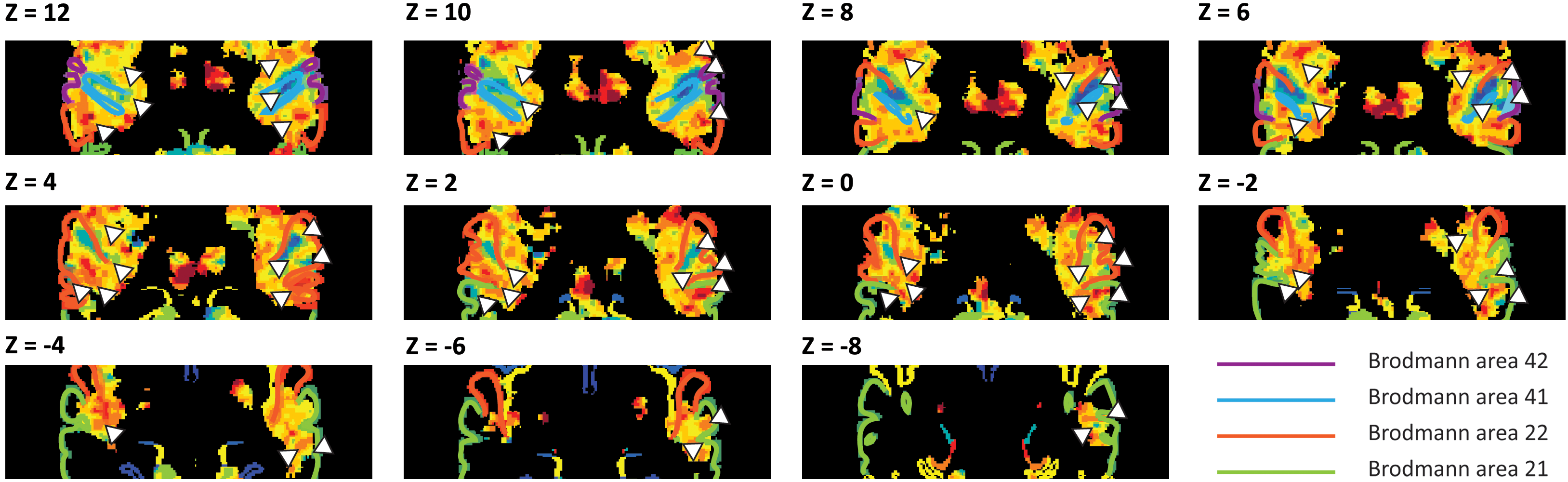

Supplement: Figure S7 — Cochleotopic maps projected on a Talairach normalized brain of Brodmann areas. Relative frequency preference maps of the averaged rising chirp group (n = 10) and falling chirp group (n = 5), within the groups' high auditory responsive areas (R>0.25, p<0.05 Bonf. Cor.). The map is presented on a depiction of the Brodmann's areas in a horizontal view. Brodmann areas 21, 22, 41, 42 are depicted upon the maps, and cochleotopic gradients' peaks are marked with white triangles. Cochleotopic gradients could be found beyond primary auditory areas (Brodmann areas 41,42) in the temporal lobe towards STS (Brodmann areas 21, 22). (TIF) [file pone.0017832.s007.tif]

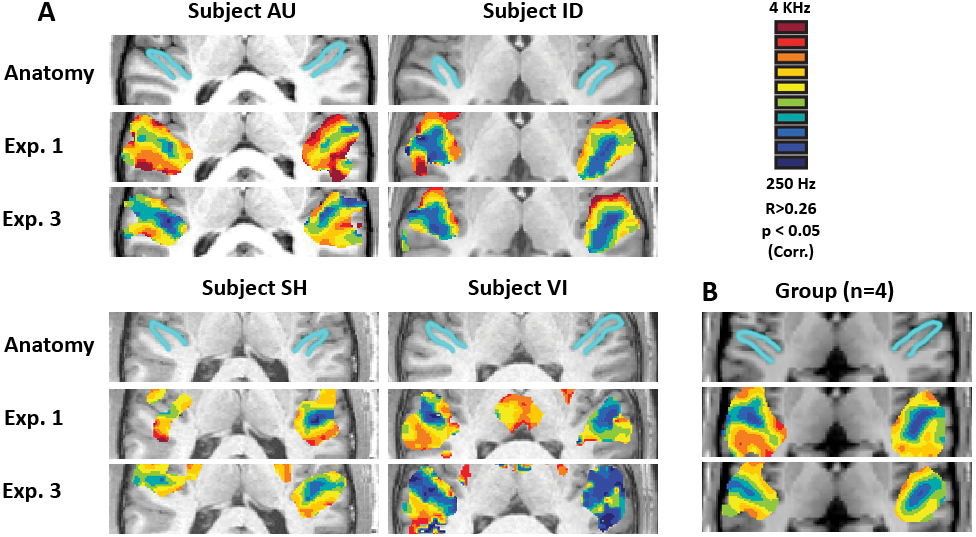

Supplement: Figure S8 — Single subject cochleotopic maps are consistent across repetitions. A. A horizontal view of the auditory cortex of 4 subjects who were scanned twice in two different days (Exp. 1 and Exp. 3) is shown, with the delineated (yellow lines) borders of Heschl's gyrus. Spectral analysis relative frequency preference maps (in individual significantly responsive areas, R>0.26, df = 299, P<0.05, corrected for multiple comparisons) are shown below. Cochleotopic maps seen on the primary auditory cortex in an anterior-posterior pattern are highly replicable across scans and across subjects. B. Group averaged maps (n = 4) for the first and second scans are presented on the MNI (Montreal Neurological Institute) standard brain, transformed to Talairach coordinates. The average maps, as well as the single subject maps, are remarkably similar in the two repeated scans. (TIF) [file pone.0017832.s008.tif]
